# Engineering Functional Biomaterials for Targeted and Localized Cancer Drug Delivery: A Structure–Property–Performance Design Perspective

**DOI:** 10.3390/ph19091498

**Published:** 2026-09-21

**Authors:** Precious Etinosa, Yusuf Olatunji Waidi, Sarah Osafo, Yiporo Danyuo, Stanley Eluu, Chukwudi Ezeala

**Affiliations:** 1 Department of Civil and Mechanical Engineering, State University of New York Polytechnic Institute (SUNY Poly), 100 Seymour Road, Utica, NY 13502, USA; 2 Department of Materials Engineering, Indian Institute of Science, C.V. Raman Avenue, Bangalore 560012, India; 3 Department of Biomaterial Sciences, Dental School, University of Ghana, Korle Bu Campus, Accra P.O. Box GP 4236, Ghana; 4 Department of Mechanical Engineering, Academic City University, Property #79-30, Agbogba, Accra GE-189-5224, Ghana; 5 Department of Biotechnology, Ebonyi State University, Enugu-Abakaliki Road, Abakaliki 480214, Nigeria; 6 Department of Materials Science and Engineering, African University of Science and Technology, Km 10 Airport Road, Galadimawa, Abuja 900107, Nigeria

**Keywords:** functional biomaterials, cancer drug delivery, structure–property–performance, targeted drug delivery, localized drug delivery, stimuli-responsive biomaterials, controlled drug release, artificial intelligence

## Abstract

**Background/Objectives:** Cancer therapy remains limited by poor tumor selectivity, systemic toxicity, biological barriers to drug transport, rapid clearance, treatment resistance, and postoperative recurrence. Functional biomaterials offer engineered systems to address these limitations through targeted, localized, controlled, and stimuli-responsive drug delivery. However, existing reviews commonly classify these systems according to carrier type, therapeutic agent, or cancer application, with less emphasis on how biomaterial structure and physicochemical properties determine therapeutic performance. This review addresses this gap using a structure–property–performance (SPP) framework to rationally engineer functional biomaterials for targeted, localized cancer drug delivery. **Methods:** The review critically examines particle size and morphology, surface chemistry and charge, targeting functionalization, stimulus responsiveness, porosity and internal architecture, mechanical stability, and degradation, and relates these design parameters to drug loading and release, systemic transport, tumor accumulation and penetration, cellular uptake, and local retention. Selected mechanistic relationships, including equations for particle diffusion, membrane wrapping, receptor-mediated endocytosis, particle aspect ratio, porous-matrix transport, implant-associated tissue diffusion, and porosity-dependent stiffness, are also discussed to support quantitative interpretation of SPP relationships. The review further evaluates these relationships across cancer-specific biomaterial applications. **Results:** The reviewed evidence shows that biomaterial properties are strongly interdependent and often involve competing requirements, so optimizing one property can improve one aspect of therapeutic performance while limiting another. Cancer-specific applications in breast, brain, colorectal, pancreatic, melanoma, ovarian, bladder, and bone cancers further demonstrate that effective biomaterial design must be matched to disease-specific biological and anatomical barriers. **Conclusions:** The SPP framework provides a design approach for moving functional biomaterials from empirical formulation toward integrated, application-specific design. Future development is expected to increasingly incorporate AI-guided and personalized design, image-guided delivery, multi-responsive functionality, and integration of cancer therapy with tissue regeneration to advance more precise and clinically translatable cancer treatments.

## 1. Introduction

Cancer comprises a diverse group of diseases characterized by uncontrolled cellular proliferation, invasion of neighboring tissues, and, in some cases, metastatic dissemination to distant organs [1]. Despite advances in prevention, diagnosis, and treatment, cancer remains a major cause of morbidity and mortality worldwide [2]. GLOBOCAN estimated ~20.6 million new cancer cases and 9.8 million cancer deaths globally in 2024, including nonmelanoma skin cancer [3]. Excluding non-melanoma skin cancer, the corresponding estimates were 19.5 million cases and 9.7 million deaths. Based on demographic changes, including population growth and aging, annual cancer incidence is projected to reach ~35 million cases by 2050, with the largest proportional increase expected in countries with lower levels of socioeconomic development [3].

Lung cancer was the most frequently diagnosed cancer and the leading cause of cancer mortality globally in 2024, followed in incidence by female breast, colorectal, prostate, and stomach cancers [3]. Cancer incidence is unequally distributed across different geographical regions [4]. In Africa, ~1.15 million new cases and 754,574 deaths were estimated in 2022 [5]. Breast, cervical, prostate, colorectal, and liver cancers contributed substantially to the incidence, whereas liver, lung, cervical, breast, and stomach cancers were the major causes of mortality [5]. In the United States, ~2.11 million new cases and 626,140 cancer deaths are projected for 2026 [6].

Population growth and aging are major demographic factors contributing to the increasing number of cancer cases. More than 25% of cancer-related deaths are attributed to preventable factors such as tobacco use, alcohol consumption, obesity, unhealthy diet, physical inactivity, environmental exposures, and carcinogenic infections [7]. Approximately 10% of cancer cases diagnosed globally in 2022 were attributable to carcinogenic infections, including *Helicobacter pylori*, human papillomavirus, hepatitis B and C viruses, and Epstein–Barr virus [1]. This burden is even higher in low- and middle-income countries, where cancer-causing infections account for ~30% of diagnosed cancer cases [8,9]. These disparities emphasize the need for effective therapeutic strategies that improve tumor selectivity, sustain therapeutic drug concentrations, and reduce cytotoxic exposure of healthy tissues.

### 1.1. Overview of Conventional Cancer Therapy

Cancer treatment is complicated by interpatient and intratumoral heterogeneity due to genetic, epigenetic, phenotypic, and microenvironmental differences [10,11,12]. Different cellular subpopulations within the same tumor type may respond differently to treatment, which enables resistant clones to survive and lead to progression or recurrence [13,14].

Major contemporary cancer treatment strategies include surgery, chemotherapy, radiotherapy, immunotherapy, molecularly targeted therapy, hormone therapy, hematopoietic stem-cell transplantation, photodynamic/hyperthermia therapy, and gene- or nucleic acid-based therapies [15]. These approaches may be used individually or in combination depending on cancer type, stage, molecular profile, treatment objective, and patient condition. Surgery remains central to the treatment of many localized solid tumors, whereas chemotherapy and radiotherapy may be used in neoadjuvant, adjuvant, or palliative care [16]. Immunotherapies enhance antitumor immune responses, while molecularly targeted therapies interfere with proteins or signaling pathways that regulate cancer-cell growth or dissemination. Hematopoietic stem-cell transplantation primarily restores blood-forming cells after high-dose chemotherapy or radiotherapy and is used mainly for hematological malignancies [17].

### 1.2. Clinical Limitations of Conventional Cancer Chemotherapy

Chemotherapy remains essential for many solid and hematological cancers. However, its effectiveness is often limited by inadequate tumor exposure and systemic toxicity. After oral or intravenous administration, a drug is expected to remain stable, avoid premature metabolism and clearance, reach the tumor, penetrate the extracellular matrix, and enter relevant cancer cells. These processes depend on the drug solubility, protein binding, metabolic stability, renal and hepatic clearance, and immune-system interactions. Consequently, only a fraction of the administered dose may reach the tumor, while healthy tissues may receive substantial exposure [18,19,20,21].

Drug distribution within solid tumors is further restricted by elevated interstitial pressure, dense extracellular matrices, necrosis, and abnormal vasculature, resulting in nonuniform drug concentrations or subtherapeutic doses [22,23,24]. Increasing the systemic dose may improve tumor exposure but is limited by toxicities affecting healthy adjacent tissues and organs [18,19]. Residual cancer cells after surgery may contribute to local or regional recurrence, while systemic therapy may not maintain effective drug concentrations within the resection site. These limitations support the development of delivery systems that improve drug stability, local retention, sustained release, tumor penetration, and therapeutic selectivity.

### 1.3. Existing Gap and Novelty/Scope of the Review

Existing reviews have provided valuable summaries of cancer nanomedicine, biomaterial classes, delivery strategies, responsive systems, and cancer-specific applications [20,21,25,26,27,28]. However, these systems are commonly organized by carrier type, therapeutic agent, stimulus, or cancer application, with less emphasis on systematic connection of material structure and physicochemical properties to therapeutic performance. This review addresses this gap using a structure–property–performance (SPP) framework. It examines how biomaterial physicochemical, architectural, mechanical, and stimuli-responsive properties regulate drug loading and release, in vivo transport, tumor retention and penetration, cellular uptake, and therapeutic efficiency.

The review first discusses the design requirements and major classes of functional biomaterials, followed by their SPP relationships and then cancer-specific applications. Then, future directions are discussed, including artificial intelligence (AI)-guided design, personalized biomaterials, image-guided localized delivery, smart multi-responsive systems, and integration of anticancer treatment with tissue repair.

## 2. Functional Biomaterial Systems for Targeted and Localized Cancer Drug Delivery

Biomaterials have traditionally been developed to restore, replace, or support damaged tissues while minimizing adverse biological reactions [29]. Advances in materials science, nanotechnology, and molecular engineering have expanded this concept toward functional biomaterials that actively interact with biological systems to improve therapeutic outcomes [30,31]. In cancer therapy, these systems provide engineered platforms for localized, targeted, and controlled drug delivery beyond conventional direct-drug administration [32].

Unlike passive carriers, functional biomaterials regulate drug delivery in response to endogenous stimuli within the tumor microenvironment, such as acidic pH, hypoxia, enzyme activity, and redox gradients, or external triggers including light, ultrasound, magnetic fields, and temperature [33]. They can also be functionalized with targeting ligands, including antibodies, peptides, aptamers, and polysaccharides, to improve tumor recognition and cellular uptake [31]. These capabilities enable precise control over the location, timing, and rate of drug release while minimizing damage to healthy tissues.

The development of functional biomaterials is driven by the limitations of conventional chemotherapy, including poor tumor selectivity, rapid systemic clearance, dose-limiting toxicity, and inadequate penetration into solid tumors [32]. By protecting therapeutic payloads, enhancing localized retention, enabling sustained release, and supporting multifunctional approaches such as imaging, immune modulation, and combination therapy, these systems improve therapeutic efficiency [34]. Their clinical performance ultimately depends on interrelated design properties, including biocompatibility, biodegradability, drug-loading capacity, controlled release, targeting specificity, and stimulus responsiveness, which provide the foundation for the engineering strategies discussed in the following section [30].

### 2.1. Essential Design Requirements and Strategies

The performance of functional biomaterials for targeted and localized cancer drug delivery depends not only on the therapeutic payload but also on the rational design of the delivery carrier [31]. An effective biomaterial must safely interact with biological tissues, protect and transport therapeutic agents, release them at the desired site and rate, and degrade without causing long-term adverse effects [34]. Achieving these objectives requires balancing competing design considerations [35], for example, increasing structural stability may prolong drug release but delay degradation, whereas maximizing drug loading can compromise formulation stability and release control [36]. Consequently, biomaterial design integrates biological compatibility, physicochemical performance, and clinical translatability [35].

#### 2.1.1. Essential Design Requirements

Biocompatibility and biodegradability are fundamental requirements for clinical translation [37]. Biomaterials should avoid cytotoxicity, inflammation, adverse immune responses, nonspecific protein adsorption, and rapid clearance while exhibiting tunable degradation synchronized with the therapeutic window to prevent premature drug release or long-term accumulation [38]. Equally important, degradation products should remain non-toxic and be readily cleared to avoid cumulative tissue damage during prolonged treatment. Natural polymers generally exhibit superior biocompatibility but limited mechanical stability and batch-to-batch variability, whereas synthetic materials offer greater control over composition and degradation but require careful optimization to ensure biological safety [39]. Thus, balancing compatibility with structural performance remains a central design challenge.

High drug-loading capacity and controlled release are equally important for maximizing therapeutic efficiency while minimizing carrier burden [37]. Drug-carrier compatibility governs encapsulation efficiency, formulation stability, and release behavior, particularly in combination therapies requiring co-delivery of agents with different physicochemical properties [40]. Effective biomaterials maintain therapeutic drug concentrations through sustained and predictable release while minimizing burst release and off-target toxicity. This is achieved by tailoring material composition, degradation kinetics, diffusion pathways, and drug-matrix interactions.

Target specificity further distinguishes functional biomaterials from conventional drug formulations. Despite extensive investigation, active targeting alone rarely overcomes the complex physiological barriers of solid tumors, including dense extracellular matrices, elevated interstitial fluid pressure, and heterogeneous vascularization [41,42]. Consequently, contemporary biomaterial design increasingly combines passive accumulation with active targeting and stimuli-responsive release to improve therapeutic precision.

#### 2.1.2. Engineering Design Strategies

To translate these design requirements into clinical performance, functional biomaterials employ complementary engineering strategies that optimize spatial, temporal, and biological control of therapy. Targeting and localization strategies achieve tumor-selective drug delivery through complementary engineering approaches, including ligand-mediated recognition, injectable depots, implantable devices, scaffolds, films, and microneedles. These platforms improve local drug retention, reduce systemic exposure, and provide the structural foundation for subsequent controlled and stimuli-responsive drug release [43,44].

Controlled-release systems regulate drug delivery by manipulating biomaterial architecture, degradation behavior, and diffusion pathways. Increasingly, these systems incorporate stimuli-responsive mechanisms that trigger drug release in response to endogenous tumor characteristics, including acidic pH, redox gradients, hypoxia, reactive oxygen species, and enzyme activity, or external stimuli such as light, ultrasound, magnetic fields, and temperature, enabling precise spatial and temporal control [45].

Modern biomaterials also support multifunctional therapies by co-delivering chemotherapeutics with genes, photosensitizers, immunotherapeutics, or molecularly targeted agents. In parallel, biomaterials have emerged as platforms for localized immune modulation through the delivery of immune checkpoint inhibitors, cytokines, vaccine adjuvants, and macrophage-polarizing agents that reshape the tumor microenvironment and enhance antitumor immunity [46].

Collectively, these design requirements and engineering strategies demonstrate that functional biomaterials extend far beyond passive drug encapsulation. Their clinical success depends on the coordinated optimization of compatibility, degradation, drug transport, targeting, controlled release, and therapeutic responsiveness, providing the engineering framework for next-generation targeted and localized cancer therapies [33,47].

### 2.2. Emerging and Innovative Functional Biomaterials

#### 2.2.1. Overview of Conventional Functional Biomaterials

First-generation functional biomaterials established the foundation for targeted and localized cancer drug delivery by improving the stability, pharmacokinetics, and therapeutic efficiency of anticancer agents compared with free drug administration. Conventional systems, including liposomes, polymeric NPs, polymeric micelles, hydrogels, implantable carriers, and microneedles, were developed to protect therapeutic payloads, prolong circulation, enhance tumor accumulation, and reduce systemic toxicity [48,49]. Liposomes achieved early clinical success through formulations such as PEGylated liposomal doxorubicin (Doxil^®^), while biodegradable polymeric carriers (e.g., PLGA, PLA, and PCL) provided greater structural stability, tunable degradation, and sustained drug release [50]. Localized systems, including hydrogels, implantable carriers, and biodegradable scaffolds, further improved therapeutic outcomes by maintaining high drug concentrations at tumor sites, whereas microneedles enabled minimally invasive transdermal delivery for superficial malignancies.

Despite these advances, conventional biomaterials remain constrained by several biological and physicochemical limitations. Most rely predominantly on passive targeting through the enhanced permeability and retention (EPR) effect, which varies considerably among tumor types and patients [51]. For example, tumor accumulation of systemically administered nanomaterials often represents only ~1–10% of the injected dose, with the remaining fraction largely distributed to clearance organs such as the liver and spleen [52]. Premature drug leakage, burst release, limited penetration into dense tumor tissues, restricted drug-loading capacity, instability during systemic circulation, and poor responsiveness to the dynamic tumor microenvironment further limit therapeutic efficiency [46]. Consequently, although these platforms transformed cancer drug delivery, their inability to integrate precise targeting, adaptive drug release, and multiple therapeutic functions has driven the development of next-generation functional biomaterials [35].

#### 2.2.2. Innovations in Functional Biomaterials

The limitations of conventional delivery systems have accelerated the development of biomaterials that actively interact with the tumor microenvironment rather than functioning solely as passive drug carriers. Advances in biomaterials science, nanotechnology, molecular engineering, synthetic biology, and computational modeling have shifted the field towards intelligent platforms capable of overcoming biological barriers, responding to disease-specific stimuli, and integrating multiple therapeutic functions [36].

Biomimetic biomaterials represent one of the most promising strategies for improving tumor targeting and immune evasion. Cell membrane-coated NPs and extracellular vesicles, particularly exosomes, exploit naturally derived biological components to evade immune recognition, prolong circulation, and enhance tumor-specific delivery. Membrane-coated NPs inherit functional membrane proteins that facilitate immune escape and, in some cases, homotypic tumor targeting [48,53]. Exosomes possess exceptional biocompatibility and can traverse biological barriers such as the blood–brain barrier (BBB) [48,53]. However, their clinical translation remains constrained by manufacturing scalability, product heterogeneity, quality control, and limited drug-loading efficiency.

In addition to biomimetic systems, protein-based, polysaccharide-based, and inorganic–organic hybrid platforms are also gaining attention as innovative biomaterials for cancer drug delivery. Protein-based nanoparticles, including albumin, ferritin, and virus-like particles, offer biocompatibility, biodegradability, self-assembly, internal drug-loading capacity, and opportunities for receptor-mediated or surface-engineered targeting [54]. Polysaccharide-based systems, including chitosan, alginate, and hyaluronic acid, are also attractive because their functional groups, biodegradability, mucoadhesion, and stimulus-responsive gelation can support controlled release, CD44-associated targeting, and injectable/localized delivery [55]. Inorganic–organic hybrid systems, such as polymer- or lipid-coated mesoporous silica nanoparticles and hybrid metal–organic frameworks, combine high surface area, tunable porosity, structural stability, and organic responsiveness, making them useful for high drug loading, gated release, imaging, and combination therapy [56]. These platforms further support the SPP framework because their performance depends on coordinated control of composition, architecture, surface chemistry, porosity, degradation, and biological-interface behavior.

To overcome the limited control of conventional carriers, molecularly engineered platforms such as metal–organic frameworks, covalent organic frameworks, DNA nanostructures, and self-assembling peptides provide greater control over drug loading, release behavior, and multifunctionality [51]. Their highly ordered architectures enable high drug payloads, stimuli-responsive release, and the integration of targeting ligands, imaging agents, and therapeutic carriers within a single platform. These programmable systems support combination therapy and gene delivery while responding to tumor-associated stimuli such as acidic pH and redox gradients [46]. Nevertheless, challenges related to structural stability, manufacturing complexity, production cost, and long-term biosafety continue to hinder clinical translation.

Molecularly imprinted polymers/materials (MIPs) are emerging synthetic-recognition biomaterials for cancer drug delivery, offering receptor-like or antibody-like molecular selectivity through engineered recognition cavities [57]. They are typically prepared by polymerizing functional monomers and crosslinkers around a template molecule, peptide epitope, drug, or biomarker, followed by template removal to generate complementary binding sites [57]. This imprinting process links material structure to performance because cavity size, shape, chemical functionality, accessibility, and crosslinking density can regulate drug loading, molecular recognition, and tumor-cell selectivity [57]. Recent MIP-based cancer-delivery systems include pH-responsive MIP carriers for controlled and sustained capecitabine release [58], doxorubicin-loaded P32 epitope-imprinted Fe_3_O_4_/polydopamine nanoplatforms for tumor-targeted chemo-photothermal therapy [59], CD73 epitope-imprinted mesoporous silica nanogatekeepers for pH/glutathione-responsive doxorubicin delivery [60], and multi-responsive sialic-acid-imprinted polymer nanocapsules designed for tumor-cell recognition and on-demand drug delivery [61]. These examples show how imprinting strategy, recognition-site architecture, crosslinking density, surface chemistry, and stimulus-responsive degradation can connect molecular recognition with drug loading, tumor selectivity, controlled release, and therapeutic performance. However, broader translation of MIPs still requires improved validation of binding selectivity in complex biological fluids, biodegradability, long-term safety, and in vivo targeting performance [57].

A further evolution is the emergence of living and biohybrid biomaterials that actively influence therapy rather than functioning solely as drug reservoirs [29,33]. By integrating synthetic biomaterials with living cells or biologically active components, these systems can sense and respond to the tumor microenvironment. Injectable hydrogels supporting CAR-T cell delivery enhance cell survival and tumor infiltration, whereas biohybrid microcarriers and cell-assisted delivery systems improve tissue penetration by overcoming barriers such as dense extracellular matrices and elevated interstitial pressure [36]. Despite their therapeutic promise, regulatory complexity, reproducibility, sterilization, storage, and maintenance of cellular viability remain major obstacles to widespread clinical application [62,63].

Complementing these material innovations, artificial intelligence (AI) is transforming biomaterial discovery from an empirical to a data-driven process. Machine learning enables rapid virtual screening of material compositions, prediction of drug-loading efficiency, optimization of NP characteristics, and modeling of pharmacokinetic behavior before experimental validation [64,65]. AI-assisted quantitative structure-activity relationship analyses, molecular docking, and predictive modeling further support the rational design of safer, more effective, and increasingly personalized biomaterial systems [66]. However, the reliability of these approaches depends on the availability of standardized, high-quality experimental datasets and continued collaboration between computational scientists, engineers, and biomedical researchers.

## 3. Structure–Property–Performance (SPP) Relationships

### 3.1. Rationale for the SPP Framework

Biological systems provide a useful rationale for understanding how physicochemical properties govern functional interactions. Viruses, antibodies, proteins, and other biological systems possess defined sizes, morphologies, surface chemistries, and hierarchical architectures that govern molecular organization, cellular interaction, and biological function. For example, viral shape, such as polyhedral, helical, or spherical morphology, influences the specific types of cells they target and the residence time. The surface chemistries of antibodies help them recognize and attach to antigens through specific noncovalent bonding. The complex physicochemical properties of proteins, surface charge, molecular size and shape govern their biological viability and function. It is evident that natural nanostructures follow physicochemical design principles [52]. It is important to understand how the physicochemical properties of nanostructures influence biological interactions and functions. This understanding will provide useful insights into how these properties can be optimized and/or integrated based on intended biological application to develop functional biomaterials. In tumor imaging, for instance, detection sensitivity does not only depend on signal-generating properties but also on efficient delivery and sufficient accumulation at the tumor site. Establishing an interaction design framework can support the rational development of nanostructures with improved targeting, delivery efficiency, and functional performance.

Functional biomaterials for cancer drug delivery can be engineered with controllable features, including size and morphology, surface chemistry, internal architecture, targeting ligands, stimuli-responsive components, and degradation behavior. However, optimization of one material property does not necessarily ensure therapeutic success. A hydrogel with high drug-loading capacity may still exhibit limited efficiency because of poor mechanical stability, uncontrolled burst release, or weak retention at the tumor bed. Accordingly, this section is focused on an SPP framework to critically examine how biomaterial design features influence physicochemical properties, interactions with biological systems, and ultimately therapeutic outcomes. Through this framework, we will identify practical design principles and structural design parameters critical to biological interactions and therapeutic performance. To provide a practical guide for the following subsections, Table 1 summarizes the major design parameters discussed in the SPP framework and links each parameter to a mechanistic or quantitative relationship, functional outcome, and therapeutic implication.

### 3.2. Size and Morphology

#### 3.2.1. Size-Dependent Transport and Cellular Uptake

Tumor accumulation generally represents only a small fraction of the injected dose, with reported values varying widely, often 1–10%, depending on particle type, tumor model, administration route, and quantification method [52]. Mononuclear phagocyte system (MPS) cells in the liver and spleen often phagocytose NPs, degrade a small fraction of them, and eventually exocytose both the degraded and intact NPs [67]. Nondegradable nanomaterials that are not efficiently cleared by the kidney may persist in tissues for months, depending on size, chemistry, degradation, and organ distribution [68]. Thus, careful engineering of nanomaterials is very important to optimize or maximize therapeutic effects, and substantial effort has been invested even as early as 1955, when a polymer-drug was reported [69].

**Figure 1:**
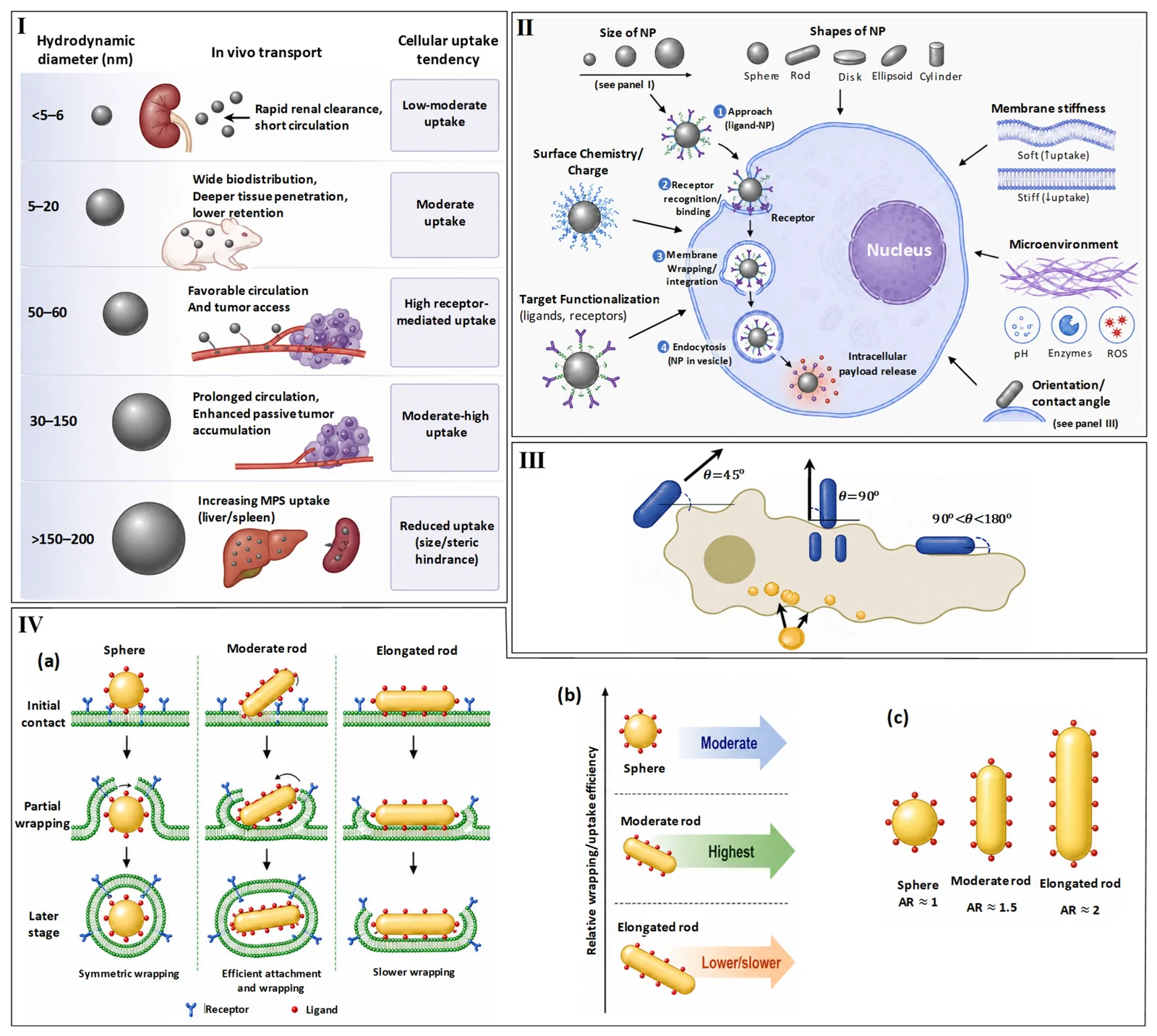
Effects of nanoparticle size, morphology, orientation, and biointerfacial properties on cellular uptake. The schematic summarizes how NP design features regulate biodistribution, tumor interaction, and endocytosis: (**I**) Different particle sizes and how they influence cellular uptake and in vivo biodistribution. (**II**) Typical cellular uptake and different contributing factors. These parameters collectively determine membrane interaction, receptor recognition, NP wrapping, and intracellular delivery. (**III**) Effect of contact angle and particle orientation on membrane wrapping of particles, with different internalization tendencies. Adapted from [81] under the Creative Commons Attribution License (CC BY). (**IV**) Effect of nanoparticle aspect ratio on membrane wrapping and endocytic uptake: (a) wrapping sequence for spherical, moderately and highly elongated nanoparticles; (b) comparison of relative uptake efficiency: moderate elongation may enhance membrane attachment and wrapping, whereas excessive elongation can slow internalization. (c) representative nanoparticle geometries with increasing aspect ratio. AR represents the approximate length-to-diameter ratio. Receptor and ligand symbols are identified within the panels.

In the engineering of nanomaterials, size and shape are perhaps the most significant determinants of effective interactions with biological tissues and ultimately optimized performance in cancer drug delivery [70,71]; see illustration in Figure 1I,II. The specific geometry features analyzed include hydrodynamic diameter, aspect ratio, curvature, and shape anisotropy. Our discussion is focused on how these features influence transport in blood, clearance by the MPS, extravasation through tumor vessels, and cellular internalization [72,73].

For approximately spherical particles, the diffusional transport in the blood or plasma can be estimated to be inversely related to the hydrodynamic size according to the Stokes–Einstein relationship [74]:

(1) $$D_{t}=\frac{k_{B}T}{3\pi \eta d_{h}}\;$$

where $D_{t}$ is the translational diffusion coefficient, $k_{B}$ is the Boltzmann constant, T is temperature, η is fluid viscosity, and $d_{h}$ is particle diameter. Although blood is not a simple Newtonian fluid, for intravenously administered NPs, Equation (1) illustrates a basic trade-off in which a decreasing particle size generally enhances diffusional mobility and interstitial penetration, whereas increasing size can improve drug payload, reduce renal loss, and sometimes enhance residence within tumor tissue [75]. In a systemic study illustrating the role of size on biodistribution [76], spherical gold NPs of different sizes (15, 50, 100, and 200 nm) were intravenously administered in mice. After 24 h, it was reported that the tissue biodistribution of the NPs strongly depended on size. The 15 nm AuNPs exhibited the highest NP levels and the broadest distribution across the evaluated tissues, including blood, liver, lungs, spleen, kidneys, brain, heart, and stomach. Notably, 15 and 50 nm AuNPs were detected in the brain tissue, indicating their ability to diffuse through the BBB under the conditions of this study, whereas 200 nm AuNPs showed only minimal diffusion.

The size dependence of cellular uptake can be explained by membrane-wrapping energetics. For a simplified spherical NP undergoing nonspecific uptake, membrane wrapping becomes thermodynamically feasible only when adhesion energy exceeds the combined bending and tension effects of membrane deformation. Assuming uniform nonspecific adhesion and negligible spontaneous membrane curvature, the lower critical radius can be expressed as [77]:

(2) $$R_{min}={\left(\frac{2B}{\alpha_{ns}-\sigma }\right)}^{\frac{1}{2}}$$

where B is membrane bending stiffness, $\alpha_{ns}$ is nonspecific adhesion energy density, and σ is membrane tension. This relation requires $\alpha_{ns}>\sigma$, but when $\alpha_{ns}=\sigma$, endocytosis is not feasible as $R_{min}\to \infty$. Additionally, membrane wrapping of NPs smaller than $R_{min}$ is not favorable, although they may still enter cells through alternative routes, including direct translocation or uptake after aggregation [78,79]. The relative contributions of membrane bending and tension during nanoparticle (NP) wrapping can be described by a characteristic length scale, $\lambda ={\left(2B/\sigma \right)}^{1/2}$ [77]. For particles with R<λ , membrane bending dominates the wrapping process, whereas tension effects become increasingly important as particle radius approaches or exceeds λ. Using representative values of $B\approx 15K_{B}T\;$ and σ≈ 0.05 mN m^−1^, Zhang et al. estimated λ to be ~50 nm [77].

For receptor-mediated uptake, ligand binding depletes receptors near the particle-membrane contact region, and the receptor diffusion from the surrounding membrane then becomes a kinetic constraint. When receptor diffusion is rate-limiting, the wrapping endocytic time was estimated by Gao et al. [80] as:

(3) $$t_{w}=\frac{\beta R^{2}}{D_{r}}$$

where $D_{r}$ is receptor diffusivity and β is a dimensionless speed factor. Assuming a constant β, Equation (3) shows that there is a quadratic relationship between the endocytic time and particle size, and under physiologically relevant parameters the optimum radius for rapid endocytosis is ~25–30 nm (50–60 nm diameter). Experimental studies summarized by Albanese et al. similarly identified an optimum receptor-mediated cellular uptake of ~30–50 nm in selected mammalian cell systems [52]. However, these ranges should not be generalized because uptake also depends on ligand density, receptor expression, cell type, membrane tension/stiffness, and endocytic pathway.

Ultrasmall NPs (<5–6 nm) are rapidly eliminated by renal clearance, whereas larger particles, particularly those >200 nm, are increasingly taken up by MPS cells in the liver and spleen. NPs of ~30–150 nm may better support prolonged circulation and passive tumor accumulation. However, 15–20 nm particles can penetrate tumor tissue more deeply but may not be retained beyond 24 h, while ~100 nm particles often remain near blood vessels because of extracellular-matrix retention. Thus, the preferred size depends on whether the design objective is circulation, tumor retention, deep intratumoral penetration, or cellular delivery.

#### 3.2.2. Shape and Orientation-Dependent Uptake

The shape of the NP is another important physical design parameter that can enhance or inhibit cellular interactions by altering local membrane curvature, particle orientation at contact, and the deformation energy required for membrane wrapping. For non-spherical NPs, the asymmetric membrane deformation makes the wrapping energy difficult to calculate analytically, particularly when membrane tension is important. Although continuum membrane models can capture large-scale deformation, incorporating lateral diffusion of lipids and receptors is computationally demanding.

To estimate the effect of shape on internalization, Huang et al. [82] used a tensionless, one-NP-thick coarse-grained membrane model to examine receptor-mediated uptake of initially upright spherocylindrical NPs. The shape was defined using an aspect-ratio parameter, $AR=L_{total}/D$, where $L_{total}$ is the total particle length and D is the particle diameter. For a spherocylindrical particle, the AR can be estimated as

(4) $$AR=\frac{l+2R}{2R}=1+\frac{l}{2R}$$

where l is the cylindrical segment length and R is the radius of the hemispherical end caps, thus AR=1 represents a sphere as l is zero. The simulations showed that uptake involves simultaneous particle rotation and membrane wrapping, as illustrated in Figure 1IV(b). A moderately elongated particle, AR=1.5, tilted slightly and was internalized relatively rapidly, whereas a more elongated particle, AR=2, is first rotated into a membrane-parallel orientation and subsequently reoriented toward near-perpendicular entry. The entry angle, θ, is defined relative to the membrane surface, where θ=0 or 180° denotes a parallel orientation and θ = 90° denotes an upright, near-perpendicular orientation.

For membrane wrapping, at fixed radius and ligand density, the simulation shows that the receptor-mediated wrapping time increases approximately with aspect ratio at high AR, because more receptors are required for complete wrapping. However, at low-to-moderate aspect ratios, this relationship is not simply proportional; a sphere (AR=1) was wrapped more slowly than a slightly elongated particle (AR=1.5) but a more elongated particle (AR=2) was wrapped slowly again, as illustrated in Figure 1IV(a,b). This indicates that moderate elongation can facilitate uptake, whereas very high aspect ratios often lead to kinetically difficult internalization due to receptor recruitment and rotational reorientation.

Therefore, the particle shape strongly influences wrapping and cellular internalization, suggesting that elongated morphologies can be particularly beneficial during circulation. In context, tubular or rod-shaped micelles can exhibit a circulation half-life that is ten times longer than that of spherical counterparts [83]. Elliptical disk particles have also been shown to be rapidly internalized (<6 min) when first contacted along their major axis, but were not engulfed after 12 h when contact occurred along the minor axis [72]. Spherical particles exhibited rapid, orientation-independent uniform uptake due to symmetry. This shape effect was independent across particle sizes tested. In another study [83], filomicelles with diameters of ~20–60 nm and 18 µm exhibited circulation half-lives of approximately five days and improved paclitaxel-mediated tumor reduction compared with free drug. Similarly, long-rod mesoporous silica NPs with an aspect ratio of ~4 exhibited longer gastrointestinal retention, delayed clearance, and greater nifedipine bioavailability than short rods or spheres of similar diameter [84].

Beyond therapy, we have shown in our LHRH-conjugated magnetite study that this principle is also relevant to TNBC imaging and diagnostics. In the study, 50 nm magnetite nanorods with an aspect ratio of ~10 produced stronger T2-weighted MRI contrast than 20 nm spherical magnetite NPs in TNBC xenografts. The nanorods showed the highest contrast-to-noise and signal-to-noise performance among the tested formulations, suggesting that anisotropic morphology can enhance diagnostic performance through altered tumor association and magnetic-field perturbation [85].

These competing geometric requirements support the development of multistage or adaptive carriers. A relatively larger or elongated structure may improve circulation and tumor retention, whereas a smaller and less anisotropic particle may improve deep tissue penetration and cellular uptake after extravasation. Overall, the available evidence supports an application-specific design rule in which small particles are generally favored for deep tissue penetration and receptor-mediated uptake. However, larger or elongated particles can be advantageous for circulation, tumor retention, vascular interaction, or imaging contrast. Rather than seeking one universally optimal geometry, biomaterial design should be aimed at matching particle size and shape to the delivery and tumor microenvironment.

### 3.3. Surface Chemistry and Charge

Surface chemistry and charge define the biological identity of functional biomaterials used for cancer drug delivery. Once an NP enters blood or interstitial fluid, its synthetic surface is quickly modified by adsorption of proteins, ions, lipids, and other biomolecules. This interfacial remodeling controls opsonization, macrophage uptake, vascular circulation, tumor penetration, receptor binding, and drug release. Therefore, the surface of a drug-delivery NP should not be viewed as a passive coating, but as a dynamic interface that mediates the transition from blood circulation to tumor localization and intracellular therapeutic action.

#### 3.3.1. Zeta Potential

The surface charge is commonly reported as the zeta potential (ζ) of NPs. As an approximate design guideline, strongly positively charged NPs (ζ > 10 mV) promote serum protein adsorption and aggregation, whereas strongly negatively charged NPs (ζ < −10 mV) undergo protein-corona-mediated phagocytic uptake [81,86]. Near-neutral NPs, often approximated as |ζ| ≤ 10 mV, generally reduce opsonization and prolong circulation [81,86]. A useful way to describe this interface is as a sequentially changing surface that optimizes NP performance. During circulation, the NP surface should be designed to be near-neutral or slightly negative and hydrophilic to be resistant to serum-protein adsorption and MPS clearance. After tumor localization or at the tumor-cell interface, however, the surface may need to undergo charge reversal to become positively charged, to become more cell-interactive and promote uptake or tumor-cell binding. Thus, the most effective surface designs should not be permanently stealthy or permanently cationic, rather, they should be adaptive surfaces that can switch from a stealth state to an active state in response to tumor-associated or intracellular cues. This ‘stealth-to-active’ transition is the central design principle that links PEGylation, protein-corona control, stimulus-responsive chemistry, and targeting functionalization.

#### 3.3.2. PEGylation and Protein-Corona Regulation

PEGylation remains one of the most widely used strategies for increasing NP circulation time. PEG chains generate a hydrated steric barrier that reduces NP aggregation, protein adsorption, opsonization, and macrophage uptake [87,88]. This effect is governed not only by the presence of PEG, but by PEG molecular weight, grafting density, chain conformation, particle size, and curvature. The PEG-chain conformation is often described using the Flory radius [88], $R_{F}\approx aN^{3/5}$, where $R_{F}$ is the Flory radius, a is the effective monomer length, and N is the degree of polymerization. The ratio between $R_{F}$ and the average distance between neighboring PEG chains, D, determines whether PEG chains form a mushroom or brush configuration: $R_{F}/D\;\leq 1$ for mushroom regime and $R_{F}/D\;>1$ for brush regime. Sparse PEG chains form a mushroom-like layer, whereas densely grafted PEG chains stretch outward into a brush layer. Suk et al. summarized data show that dense PEG coatings provide stronger surface shielding with reduced macrophage uptake and improved blood circulation [88]. However, Walkey et al. showed that PEGylation does not simply eliminate protein adsorption. Using 15, 30, 60, and 90 nm gold NPs grafted with 5 kDa PEG at different densities, they showed that increasing PEG density reduced total serum-protein adsorption by 94–99% relative to ungrafted particles, but adsorption remained statistically significant even at the highest PEG densities. This finding is important because it shows that PEG reduces the protein corona but does not eliminate it [89].

### 3.4. Stimulus-Response Design

Functional biomaterials with stimulus-responsive surface chemistry provide drug-delivery systems that respond to tumor-associated biological cues or external triggers. Rather than relying only on passive diffusion, these systems use cues to induce PEG shedding, charge reversal, photodynamic activation, or drug release. A typical key design concept is to ensure that the NP surface remains stable during circulation, becomes more interactive within tumor tissue, and releases the drug after cellular uptake.

A clear demonstrative example is a pH-responsive system, illustrating this sequential design. Because tumors and intracellular vesicles provide acidic environments, acid-sensitive surfaces can be designed to protonate ionizable groups, detach PEG, expose ligands, or release drugs through acid-labile bonds [87]. For example, Amoozgar and Yeo described hydrazone-based PEG shedding systems in which PEG is retained at physiological pH but removed under acidic conditions, exposing a more cell-interactive surface [90]. Du et al. provided a more precise dual-pH model using polymer-doxorubicin NPs that remained negatively charged at pH 7.4, converted to a positive surface at tumor extracellular pH of ~6.8, and then released DOX through hydrazone cleavage at endosomal/lysosomal pH of ~5.0. The particles were ~27 nm, contained 8.32 wt% DOX, and showed 76% conjugation efficiency. Their zeta potential became positive within 10 min at pH 6.8 but remained negative for at least 2 h at pH 7.4, while DOX release after 184 h was only about 22.60% at pH 7.4 and 25.56% at pH 6.8, but exceeded 75% at pH 5.0 [91].

The key design insight is that pH responsiveness should not be limited to release alone. In another systematic study, extracellular acidity was shown to improve internalization, while stronger intracellular acidity triggered drug release. This was supported by stronger uptake in MDA-MB-231 cells at pH 6.8 than at pH 7.4, followed by nuclear DOX accumulation over 6–24 h. In drug-resistant SK-3rd cancer stem cell spheres, it was demonstrated that dual pH-sensitive NPs inhibited tumor sphere progression more effectively than free DOX or non-cleavable controls under acidic conditions [91].

Beyond internal biological cues, external stimuli such as heat, magnetism, ultrasound, and light provide control over release location, dosing, and timing. Thermoresponsive nanocarriers retain the drug at body temperature but rapidly release it in locally heated tumor tissue at a suitable elevated in vivo temperature. Thermosensitive liposomes, including doxorubicin-loaded ThermoDox, represent one of the most clinically advanced examples [87].

One thermoresponsive liposomal mechanism uses ammonium bicarbonate decomposition to generate gas bubbles that disrupt the lipid bilayer: NH_4_HCO_3_(aq) → NH_3_(aq) + H_2_O(l) + CO_2_(g). This produces membrane defects, accelerates drug release, and can improve ultrasound imaging contrast. Magnetic systems can provide either magnetic guidance or alternating-magnetic-field-induced heating. Ultrasound systems use cavitation, acoustic droplet vaporization, or mechanical destabilization to enhance release and cellular uptake.

Light systems can trigger photocleavage, photoisomerization, photothermal heating, or ROS generation, which has been exploited in cancer treatment. For example, Li et al. reported that NIR/ROS-responsive photodynamic systems increased cancer-cell apoptosis by a factor of 5 and achieved 100% mouse survival at 50 days, whereas CpG alone reached 0% survival even at earlier time points [92]. In another study, Meng et al. developed ~78 nm core–shell NPs containing chlorin e6, docetaxel, and anti-Twist siRNA, with DSPE-PEG5000 used to screen the positive polymeric carrier and improve circulation. Upon irradiation, chlorin e6 generated singlet oxygen, enabling photodynamic treatment near the illuminated surface tumor regions and also enhanced drug release in deeper tumor regions; Ce6 + hν → Ce6* and Ce6* + O_2_ → ^1^O_2_. Compared with free drugs, the NPs increased intratumoral exposure to docetaxel and anti-Twist siRNA by ~2.5- and 2-fold, respectively. The NPs also suppressed both primary TNBC growth and lung metastasis by more than 80% [93].

### 3.5. Target Functionalization

Target functionalization is typically aimed at increasing selective interaction between NPs and malignant cells by treating the surface with antibodies, peptides, aptamers, affibodies, or small molecules. However, there is a critical caveat, in vitro receptor binding does not always guarantee cancer-cell delivery in vivo [94,95]. For example, Dai et al. [96] showed that PEGylated, Trastuzumab-coated gold NPs specifically bound ErbB2-positive SKOV-3 cells in vitro, but after intravenous administration, only a very small fraction reached cancer cells. Instead, most of the NPs were trapped in acellular tumor regions or taken up by tumor-associated macrophages. Across different tumor models, folate-functionalized NPs also failed to significantly improve cancer-cell delivery, and Trastuzumab-functionalized silica NPs showed similar limitations [96].

Therefore, targeting functionalization or a ligand should not be evaluated only by receptor affinity or cell-culture uptake, it should also overcome serum adsorption, macrophage uptake, vascular extravasation, and extracellular-matrix diffusion. Dai et al. [96] showed that tumor-associated macrophages can dominate NP interactions even when targeting ligands are present, and it was shown that silica NPs interacted with the macrophages 30 times more than cancer cells. Therefore, active targeting should be understood as a late-stage event that can only occur after the NP has survived earlier biological barriers.

Another relevant concept involves shielding ligand presentation from uncontrolled biocorona formation, as demonstrated by Oh et al. [97]. Their GST-HER2-affibody protein corona shield reduced serum-protein adsorption while preserving HER2-specific uptake. The system was also modular, since an EGFR-binding affibody could be substituted to target EGFR-positive MDA-MB-468 cells. This supports the idea that targeting ligands require controlled orientation and a corona-resistant interfacial environment, not merely chemical attachment to the NP surface. In another study, Mamnoon et al. provide a useful tumor-penetrating targeting example for TNBC. Their iRGD-conjugated polymersomes first exploited the RGD motif to interact with αvβ3 and αvβ5 integrins, then subsequent cleavage exposed the CendR motif, which binds NRP-1 and promotes endocytosis or transcytosis [98]. This is important because iRGD adds a penetration function to receptor recognition, which addresses a limitation of ligands that bind tumor cells but do not necessarily carry the nanocarrier deeply into solid tumor tissue.

Obayemi et al. conjugated [D-Lys^6^]-LHRH to prodigiosin and paclitaxel for TNBC targeting [99]. LHRH receptors were more highly expressed on MDA-MB-231 cells and tumor tissue than on non-tumorigenic breast epithelial cells. siRNA knockdown reduced LHRH-R mRNA by ~70% and 90% and diminished the enhanced activity of the conjugated drugs, supporting receptor-mediated targeting. LHRH conjugation also increased drug-tumor adhesion. Specifically, PGS-LHRH adhesion forces increased to ~51.1, 86, and 101 nN at early, mid, and late tumor stages, compared with 21, 29, and 26 nN for unconjugated PGS. Two weekly 10 mg/kg doses of LHRH-conjugated PGS or PTX eliminated early-stage xenografts and produced substantially greater shrinkage of established tumors than the unconjugated drugs, without major liver, lung, or kidney toxicity. This study links targeting functionalization to receptor dependence, interfacial adhesion, tumor response, and off-target safety.

The most promising targeting designs are therefore stimulus-controlled systems in which ligands remain shielded during circulation and are unmasked only after exposure to tumor-associated cues, or in which localized biomaterial release delivers receptor-targeted drugs directly to residual tumor cells. In this design framework, the carrier first maintains a stealth surface to prolong circulation or local retention, then accumulates or remains at the tumor site, undergoes surface activation, promotes cancer-cell uptake, and finally triggers intracellular or local drug release.

### 3.6. Porosity, Internal Architecture, and Degradation

Within the SPP framework, pore dimensions, pore arrangement, and bulk architecture are structural variables that determine the transport and mechanical properties of a drug-delivery material. Pore size and connected pore volume determine how efficiently fluids and therapeutic molecules diffuse and move through the material, while internal surface area determines the space available for drug adsorption and interaction with the matrix. These properties control drug loading, effective diffusion, release duration, degradation, and structural stability, which ultimately determine local drug exposure, tissue interaction, and therapeutic efficiency [100,101].

Porosity is commonly expressed as the ratio of pore volume to the total material volume. According to the IUPAC pore-width classification, micropores have widths not exceeding ~2 nm, mesopores range from ~2 to 50 nm, and macropores exceed ~50 nm [102]. However, neither total porosity nor nominal pore size alone adequately predicts material performance, because only pores that are open, accessible, and interconnected with the surrounding fluid contribute effectively to drug loading and molecular transport. Consequently, two biomaterials with similar total porosity or average pore size may exhibit different release profiles if their pores differ in orientation, connectivity, constrictivity, or effective diffusion distance [103,104].

The movement of a drug through a porous material can be described using an effective diffusion coefficient

(5) $$D_{eff}=D_{0}\frac{\varepsilon_{c}\delta }{\tau }$$

where $D_{0}$ is diffusion in the free liquid, $\varepsilon_{c}$ is the connected porosity, δ is the constrictivity which accounts for restrictions at narrow or partly blocked pore openings, and τ is the diffusive tortuosity factor which represents the increase in diffusion distance caused by indirect transport paths. Equation (5) shows that increasing porosity may improve diffusion, whereas transport reduces if the pores are poorly connected or if there are tortuous pathways [104]. However, most studies report only average pore size, total porosity, or general architecture without relating these parameters to performance. The following discussion examines how porosity and internal architecture govern behavior and therapeutic performance across different biomaterial systems.

#### 3.6.1. Hydrogel Mesh and Macroporous Architecture

Hydrogels exhibit two characteristic transport scales with distinct functions. Lu et al. reported polymer-network mesh sizes of ~5–500 nm and larger engineered pores of ~10–500 µm [105]. The nanoscale mesh primarily regulates the diffusion of dissolved drugs and proteins, whereas the larger pores facilitate cell migration. Increasing polymer concentration or crosslink density generally reduces the hydrogel mesh size, thereby restricting drug diffusion. These changes may also increase stiffness and reduce swelling, both of which can further change release behavior [106,107]. Thus, the effects of hydrogel architecture on drug release are inherently connected with polymer chemistry, swelling, and mechanical properties.

Drug size and drug-polymer interactions determine the extent to which the hydrogel network regulates release. Woodring et al. noted that molecules smaller than the hydrogel mesh can diffuse through the hydrated matrix, whereas larger molecules may require network swelling or degradation before release [106]. The interaction behavior was demonstrated by Liu et al. using an acid-sensitive paclitaxel-gemcitabine hydrogel, which degraded by approximately 97% within one week at pH 5.8. Nearly all gemcitabine was released within three days, whereas paclitaxel release continued for approximately one week [108]. Despite being incorporated into the same network, hydrophilic gemcitabine diffused rapidly through the aqueous phase, while hydrophobic paclitaxel was retained longer through stronger interactions with the material. Thus, mesh size defines the available transport pathway, whereas drug solubility and drug-polymer interactions control the rate of transport.

Stimulus-responsive degradation provides another way to connect hydrogel architecture to therapeutic performance. Wang et al. showed that a ROS-responsive PVA-TSPB hydrogel degraded more rapidly in the ROS-rich tumor microenvironment, thereby increasing the local release of gemcitabine and anti-PD-L1 and improving tumor suppression in B16F10 and 4T1 mouse models [109]. In contrast, Song et al. used a 3D porous PEGylated poly(L-valine) hydrogel to sustain the release of tumor-cell lysate antigens and the TLR3 agonist poly(I) for more than one week. This prolonged release promoted dendritic-cell recruitment to draining lymph nodes, stimulated cytotoxic T-cell responses, and suppressed tumor growth [110]. Lv et al. similarly incorporated doxorubicin and immunostimulatory cytokines into a PELG-PEG-PELG hydrogel, producing an initial burst followed by sustained release and enhancing the chemoimmunotherapeutic response against melanoma [111]. Collectively, these studies show that hydrogel performance is governed by the combined effects of pore and mesh architecture, drug-polymer affinity, and the rate and mechanism of network degradation.

#### 3.6.2. Porous and Fibrous Scaffolds

In bulk scaffolds, pore architecture governs both drug transport and biological integration. Large, interconnected pores control infiltration of nutrients, immune cells, and newly formed tissue, but increasing pore volume reduces the solid material available for mechanical support. Zielińska et al. identified porosities above 90% and pore diameters of ~300 µm as a broadly favorable range for tissue-engineering scaffolds [112]. However, these values should not be taken as a general optimum range because the required architecture depends on the target tissue, cell type, scaffold thickness, and mechanical demands.

Obayemi et al. demonstrated this structure-performance relationship using PLGA-PEG and PLGA-PCL scaffolds for localized paclitaxel and prodigiosin delivery [113]. Both scaffolds retained highly interconnected pores of ~88–109 µm and porosities of 88–94% after drug loading. Despite their similar pore architectures, the PLGA-PEG scaffolds released ~85.5% of prodigiosin and 81.5% of paclitaxel over 56 days, compared with 73.5% and 66.0%, respectively, from PLGA-PCL. The faster release from PLGA-PEG was attributed primarily to greater water penetration through the more hydrophilic PEG-containing matrix, whereas PCL limited water uptake and slowed both drug release and degradation. Thus, pore architecture defines the available transport network, while polymer chemistry controls fluid access and the rate at which that network changes.

Eluu et al., extending this targeting mechanism to nondegradable microporous implants, developed a PDMS device with a drug-loaded PNIPA hydrogel core for localized delivery of paclitaxel, prodigiosin, and their LHRH-conjugated forms [114]. The elution profile over 30 days at 37 °C shows that ~72% and 70% of paclitaxel and PTX-LHRH were released, compared with 33% and 29% of prodigiosin and PG-LHRH, respectively. In a related study [115], they incorporated 5 wt% magnetite NPs into the PDMS matrix and increased the 30-day release at 37 °C to ~93.1% and 85.7% for paclitaxel and PTX-LHRH and 39.8% and 39.6% for prodigiosin and PG-LHRH, respectively. Together, these studies show that the porous structure provides a sustained local transport pathway, whereas drug physicochemical properties and matrix modification regulate the release rate. However, because pore size, connected porosity, and permeability were not quantitatively correlated with release, the independent contribution of pore architecture remains unresolved. This represents an important limitation in the current literature and highlights the need for future studies to directly correlate pore size, connected porosity, permeability, and degradation-dependent architectural changes with drug-release kinetics and therapeutic performance.

#### 3.6.3. Mesoporous Silica and Hollow Nanoarchitecture

Unlike bulk scaffolds, mesoporous silica contains nanoscale channels of ~2–50 nm that function as drug reservoirs and diffusion pathways rather than spaces for cell infiltration [116]. Performance depends on pore diameter, channel structure, hollow volume, surface chemistry, and pore coatings [117].

Gao et al. studied hollow mesoporous silica NPs with pore diameters of 3.2, 6.4, and 12.6 nm [118]. DOX-loading efficiencies reported were 59.6%, 50.1%, and 65.1%, respectively, showing that loading was not controlled by pore size alone. The hollow volume, accessible surface area, and drug-silica electrostatic interactions also contributed. In contrast, release generally increased with pore diameter because larger pores reduced transport resistance. For 12.6 nm pores, ~4.5% of DOX was released at pH 7.4 within 30 h, compared with ~35% at pH 5.0. The 72 h IC_50_ in MCF-7/ADR cells decreased from 8.7 to 5.7 and 3.5 µg/mL as pore size increased from 3.2 to 6.4 and 12.6 nm, respectively, indicating greater intracellular release. This advantage remains application-specific, as smaller pores may be preferable when prolonged retention and minimal premature leakage are required.

Internal surface chemistry also affects loading. Meng et al. reported decreasing DOX-loading capacities of 8.4%, 4.2%, 1.2%, and 0.1% at pH 7.4 in mesoporous silica modified with phosphate-, carboxyl-, hydroxyl-, and amine-groups, respectively [119]. These differences show that high internal surface area improves loading only when the surface chemistry provides favorable drug-surface interactions. Responsive pore coatings can further regulate drug release. Saroj et al. synthesized a poly(acrylic acid)-coated etoposide system that released 85% of the drug at pH 5.6, 70.72% at pH 6.8, and 36.21% at pH 7.4 [120]. In this design, the mesopores provided storage capacity and diffusion pathways, whereas the pH-responsive coating regulated pore accessibility and thereby controlled release under acidic conditions.

#### 3.6.4. Architecture and Implantable Drug-Delivery Systems

In implantable drug-delivery systems, the spatial arrangement of pores, layers, fibers, and drug-rich regions determines the diffusion distance between the drug and surrounding tissue, thereby influencing fluid infiltration, degradation, and release [121]. Qian et al. modified PLGA millirods coated with membranes containing 10–50 wt.% NaCl or 5–20 wt.% PEO, and reported that dissolution of these water-soluble additives created pores and sustained drug release for up to five weeks [122]. Layered millirods further combined an outer DOX/PEO burst-release layer with a slower PLA/PEG layer, producing an initial loading dose followed by sustained release for approximately ten days. These studies show that controlled pore formation and layer positioning can separate rapid and prolonged release phases.

Drug transport from an implant into surrounding tissue can be described by Fick’s diffusion-elimination equation, with first-order elimination

(6) $$\frac{\partial C}{\partial t}=D_{t}\nabla^{2}C-\gamma C$$

where C is tissue drug concentration, $D_{t}$ is the tissue diffusion coefficient, and γ is the first-order elimination constant. Equation (6) shows that local drug distribution depends not only on release from the implant but also on diffusion and clearance within the surrounding tissue. Weinberg et al. reported that therapeutic concentrations often extended only a few millimeters from the implant, while fibrous encapsulation further restricted transport. Thus, the host response becomes part of the effective delivery architecture.

Dong et al. combined two physical forms of β-lapachone within the same PLGA millirod to produce a two-stage release profile [123]. The formulation contained 31 wt.% β-lapachone/hydroxypropyl-β-cyclodextrin complex, 19 wt.% non-complexed β-lapachone, and 50 wt.% PLGA. The more soluble cyclodextrin-complexed fraction produced a burst release of ~0.5 mg within 12 h, whereas the non-complexed drug, which formed a molecular-level mixture with PLGA, provided sustained release at ~0.01 mg/day over the following 22 days. This result shows that the physical state and polymer association of a drug can be used to program multiphase release, even though implant porosity was not characterized.

In another study, Li et al. combined pore architecture and layer positioning in a dual-drug implant [124]. Two outer 3D-printed gelatin/alginate scaffolds containing MSA-2 enclosed an internal DOX-loaded PLGA fiber patch. The outer scaffolds had pores of 10–60 µm, whereas the PLGA fibers were 200–800 nm in diameter. Greater fluid access at the implant surface promoted early MSA-2 release, while the inner PLGA fibers and surrounding layers delayed DOX release. Reversing the layer sequence reduced therapeutic efficiency, confirming a direct structure-performance relationship. The optimized architecture increased intratumoral IFN-β from 7.2 to 26.1 ng/g within 12 h, M1 macrophage polarization from 18.4% to 72.0%, and CD8^+^ tumor-infiltrating T cells from 7.4% to 42.1%. Thus, performance depended not only on co-delivery of the two agents, but also on their spatial arrangement and the resulting release sequence.

#### 3.6.5. Mechanical Behavior and Degradation

Porosity enhances water infiltration and molecular transport but reduces the solid material fraction available for mechanical support. This trade-off can be described using the Gibson–Ashby power-law relationship for cellular solids [125], in which the relative elastic modulus scales with relative density. Since relative density can be expressed as the solid volume fraction, $\rho_{p}$/$\rho_{s}=1-\varepsilon ,\;$the relationship can be written as

(7) $$\frac{E_{p}}{E_{s}}=C{\left(1-\varepsilon \right)}^{n}$$

where $E_{p}$ and $E_{s}$ are the elastic moduli of the porous and solid materials, respectively, ε is porosity, and C and n are architecture-dependent constants. Equation (7) shows that stiffness generally decreases with increasing porosity, although the magnitude of this reduction depends on pore geometry and connectivity. This relationship is most applicable to porous solids or scaffolds, whereas hydrogel mechanics may require additional consideration of crosslink density, swelling, and polymer-solvent interactions.

The Obayemi scaffolds exhibited kPa-scale mechanical properties [99]. PLGA-PCL scaffolds were generally stiffer than PLGA-PEG scaffolds but also showed slower drug release and degradation. Thus, the polymer composition and architecture that improved mechanical stability also restricted water penetration and delayed release, illustrating the trade-off between structural integrity and transport performance.

Degradation further changes the pore architecture during treatment. Obayemi et al. reported ~9–18% mass loss during the first three weeks, followed by losses of up to ~85% as polymer hydrolysis and erosion accelerated. This structural evolution increased fluid access, enlarged transport pathways, and changed the drug-release mechanism.

A similar architecture-degradation relationship has been observed in mesoporous silica. Qiao et al. described rapid initial degradation of MCM-41 in simulated body fluid, followed by slower dissolution after formation of a calcium/magnesium silicate layer, with nearly complete degradation after ~two weeks at 0.5 mg/mL [126]. Li et al. also reported approximately 30%, 70%, and 90% degradation for silica systems with surface areas of 282, 829, and 958 m^2^/g, respectively, within 2–4 h [127]. These results suggest that greater fluid-accessible surface area can accelerate silica degradation, although framework composition and surface chemistry also influence degradation. Mechanical properties and degradation should therefore be considered together with pore architecture. The initial structure determines fluid access and mechanical stability, whereas degradation continuously changes that structure. To summarize these design trade-offs, Table 2 compares how porosity, architectural, degradation, and mechanical design parameters influence therapeutic goals, advantages, and performance limitations in localized cancer drug-delivery biomaterials.

## 4. Biomaterial Cancer-Type Specific Applications

Functional biomaterials can be engineered to address biological barriers and therapeutic needs of specific cancers by controlling their architecture, structure, surface functionality, mechanical behavior, and release kinetics. In this section, cancer-specific examples are discussed through the SPP concept, emphasizing how material design features influence tumor targeting, localized retention, and therapeutic outcome.

The depth of discussion across cancer types reflects both the scope and focus of this review and differences in the volume, diversity, and translational maturity of available biomaterial-based drug-delivery literature. The selected examples emphasize studies that clearly connect biomaterial design variables to SPP outcomes such as systemic transport, tumor penetration, cellular uptake, local retention, controlled release, and/or therapeutic performance. Therefore, the following subsections are intended to illustrate how the SPP framework applies across diverse tumor contexts while recognizing that the type and maturity of available evidence vary by cancer type.

### 4.1. Breast Cancer

Breast cancer remains one of the most common malignancies among women worldwide and is driven by genetic, environmental, lifestyle, and biological factors [128]. Although different methods, such as surgery, chemotherapy, or radiotherapy, have improved certain outcomes, recurrence, metastasis, drug resistance, tumor heterogeneity, and immune evasion remain major challenges. Functional biomaterial-based strategies are therefore being developed to optimize localized delivery and tumor targeting.

Postoperative recurrence is a major clinical concern and illustrates the importance of macroscale architecture and local retention. To address this, an R837-loaded niobium carbide MXene-modified biodegradable 3D-printed scaffold (BG@NbSiR) was designed to eliminate residual tumor cells, suppress metastasis, and induce long-term immune memory in breast cancer models [129]. Single-cell RNA sequencing showed broad modulation of tumor- and immune-related pathways, suggesting remodeling of the postoperative tumor microenvironment. Similarly, a sprayable thermosensitive chitosan hydrogel loaded with melittin/E15 NPs reduced recurrence, promoted wound healing, reversed immunosuppression, and enhanced anti-PD-L1 therapy [130].

At the nanoscale, surface structure and loading strategy strongly influence receptor-mediated delivery. For example, ferritin nanocarriers loaded with vandetanib or lenvatinib selectively killed folate receptor-positive MCF-7 and T47-D cells but caused no toxicity to healthy breast epithelial cells [131]. Passive loading performed better than pH-dependent ferritin reassembly because it preserved drug integrity and carrier stability. In another approach, neem-derived extracellular vesicles functionalized with chitosan and PEGylated graphene oxide delivered ERα-targeting siRNA to MCF-7 cells through CD44-mediated recognition [132].

**Figure 2:**
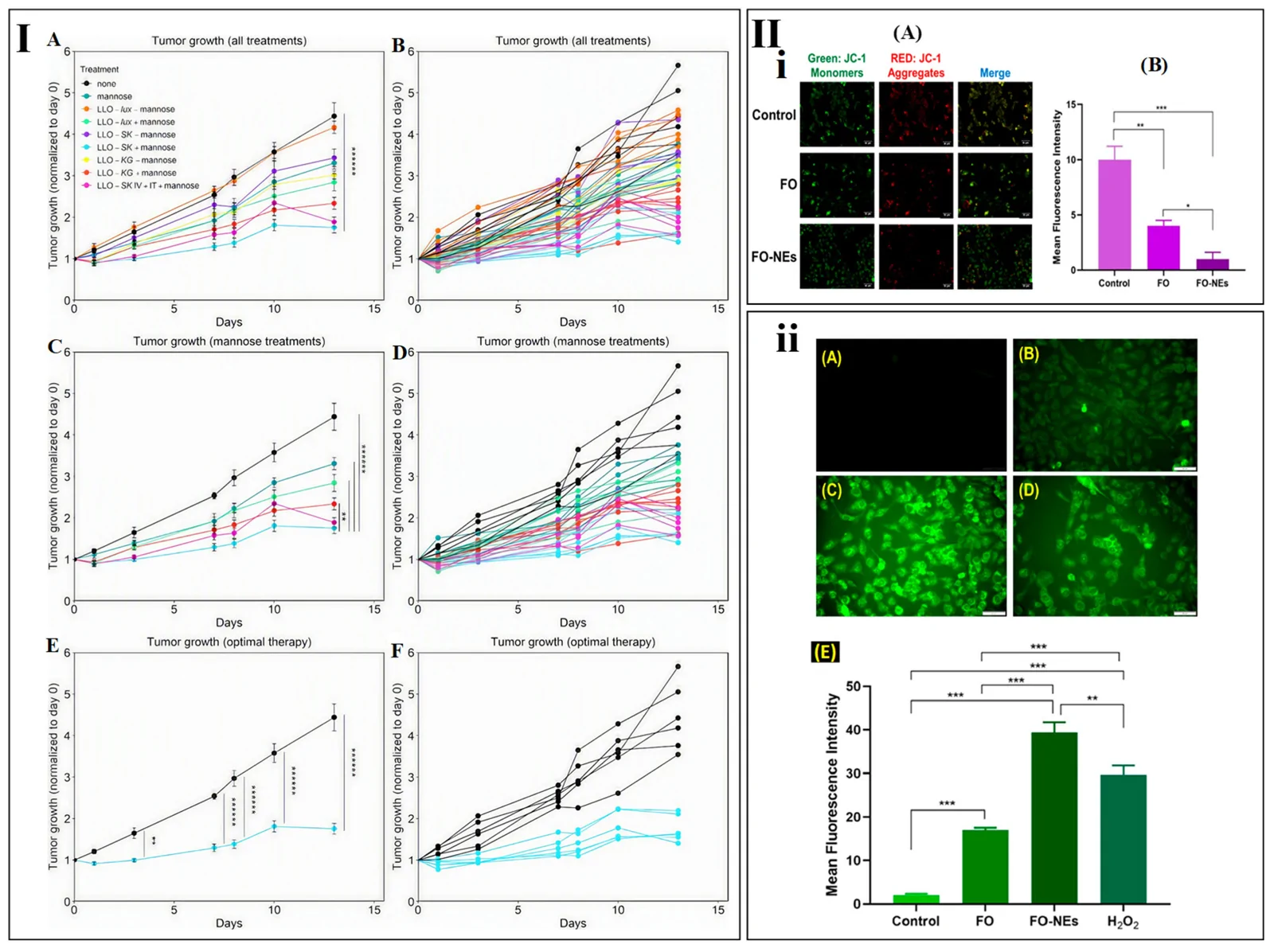
Multifunctional nanoplatforms and bioengineered systems for breast cancer therapy. (**I**) Immunomodulatory bacterial therapy: Tumor growth kinetics for all treatment groups (A,B), mannose-treated cohorts (C,D), and control versus LLO-SK-treated mice (E,F). IPTG was administered in all bacterial treatment groups. Data are mean ± SEM (*n* = 6); * *p* < 0.05, ** *p* < 0.01, *** *p* < 0.001 and ****** *p* < 0.000001. Rep. from [133], under the Creative Commons Attribution License (CC BY). (**II**) (i) (A) Effect of free FO and FO-loaded nanostructures (FO-NEs) on mitochondrial membrane potential (MMP) in MDA-MB-231 breast cancer cells, (B) with corresponding mean fluorescence intensity (MFI) quantified from red JC-1 aggregate fluorescence (scale bar = 50 µm). (ii) Intracellular reactive oxygen species (ROS) levels in (A) healthy cells, (B) free FO-treated cells, (C) FO-NE-treated cells, and (D) H_2_O_2_-treated cells (positive control), with (E) corresponding MFI quantification. Rep. from [143], under the Creative Commons Attribution License (CC BY).

As a complementary immunomodulatory strategy, engineered bacterial endosymbionts have been shown to reprogram macrophages and modulate the tumor microenvironment, resulting in reduced TNBC tumor growth in vivo [133] (Figure 2I). Ligand density, receptor affinity, and stimulus-responsive chemistry are important interfacial design features for receptor-mediated breast cancer delivery. LHRH-R is reported in ~50–60% of breast cancers, making it a useful target for ligand-guided delivery [134]. The engineered LHRH-III′ peptide showed more than sixfold higher internalization than [D-Lys^6^]-LHRH-I while retaining strong receptor affinity [135]. When conjugated to camptothecin through a glutathione-responsive disulfide linker, the peptide-drug conjugate improved antitumor activity while reducing hepatic accumulation to about one-sixth of the control peptide. Similarly, an LHRH-targeted protein-nanodiamond nanocomposite co-delivering doxorubicin and dasatinib achieved >80% drug-loading efficiency and pH-responsive release, with ~65% drug release after 72 h under acidic tumor-like conditions compared with <20% at physiological pH [136]. This translated into enhanced activity against drug-resistant breast cancer cells, reducing the IC_50_ of doxorubicin to 45.63 ng/mL in MDA-MB-231 cells and dasatinib to 35.85 ng/mL in MDA-MB-468 cells, while producing 55% tumor-growth reduction and 100% survival after 44 days in treated mice.

Triple-negative breast cancer (TNBC) further highlights the need to match biomaterial properties to tumor biology. Because TNBC lacks estrogen receptor (ER), progesterone receptor (PR), and HER2 expression, biomaterial systems often exploit alternative features such as hypoxia, altered metal-ion levels, dysregulated signaling, and folate receptor expression [137]. Peptide-based supramolecular hydrogels used self-assembled architecture to induce caspase-3/7-mediated apoptosis in MDA-MB-231 and MDA-MB-468 cells with limited toxicity toward HEK293 cells [138]. Folic acid-functionalized gold nanocluster-PLGA nanocarriers combined ligand targeting, polymeric encapsulation, and gold-cluster functionality to deliver gliotoxin, achieving IC_50_ values of 407 nM and 218.7 nM in MDA-MB-231 and MDA-MB-468 cells, respectively, while downregulating HIF-1α and Notch signaling [139]. An injectable RAFT-synthesized triple-responsive polymeric nanocarrier further integrated Cu^2+^-guided tumor recognition, ROS-mediated apoptosis, and nucleic-acid delivery [140].

Controlled-release nanocarriers show how internal structure and stimulus responsiveness regulate therapeutic exposure. Halloysite nanotube-based CMC/PEG hydrogels provided 46% 5-fluorouracil entrapment efficiency and 87% loading efficiency, with enhanced release under acidic tumor-like conditions and selective cytotoxicity toward MCF-7 cells [141]. Poly(salicylic acid-co-phenylalanine) NPs enabled paclitaxel/PF543 co-delivery while also contributing intrinsic anticancer activity [142]. Similarly, optimized frankincense oil nanoemulsions with an average particle size of 65.1 ± 4.21 nm enhanced anticancer efficacy against drug-resistant MDA-MB-231-TR cells, reducing the IC_50_ from 22.5 µg/mL for free oil to 13.2 µg/mL by increasing ROS generation and mitochondrial dysfunction (Figure 2II). Other systems used particle-size control, redox-sensitive chemistry, magnetic hydroxyapatite composition, or nanogel architecture to improve drug retention, release control, photodynamic activity, or activity against resistant cells [143,144,145,146].

Beyond direct cytotoxicity, bioengineered systems are being developed to remodel the immune microenvironment. Genetically engineered *Bacillus subtilis* endosymbionts delivered intracellular transcription factors, reprogrammed macrophages, altered cytokine profiles, reshaped immune-cell populations, and reduced TNBC growth in vivo (Figure 2I) [133]. Lipid-encapsulated bacterial endosymbionts further sustained intracellular ATP production for up to 60 h under nutrient-deprived conditions, promoted pro-inflammatory macrophage polarization, and suppressed breast cancer progression and metastasis [147].

### 4.2. Glioblastoma

Glioblastoma (GBM) is an aggressive WHO grade 4 brain tumor characterized by diffuse invasion, postoperative recurrence, immunosuppression, and poor drug access across the BBB [148]. Functional biomaterials for GBM therefore aim to solve two linked design problems: maintaining high local drug exposure after surgery and enabling targeted delivery across the BBB for infiltrative or unresectable disease.

Postoperative recurrence highlights the value of injectable and implantable depots. A dual-sensitive thermosensitive hydrogel containing ROS-responsive NPs that is co-loaded with bis(2-chloroethyl) nitrosourea (BCNU) and temozolomide (TMZ) was reported to provide a sustained local release after >90% tumor resection and extended median survival to 65 days, nearly twofold longer than surgery alone [149]. This illustrates how hydrogel injectability, thermal gelation, ROS responsiveness, and combination-drug loading can convert the resection cavity into a localized therapeutic reservoir.

**Figure 3:**
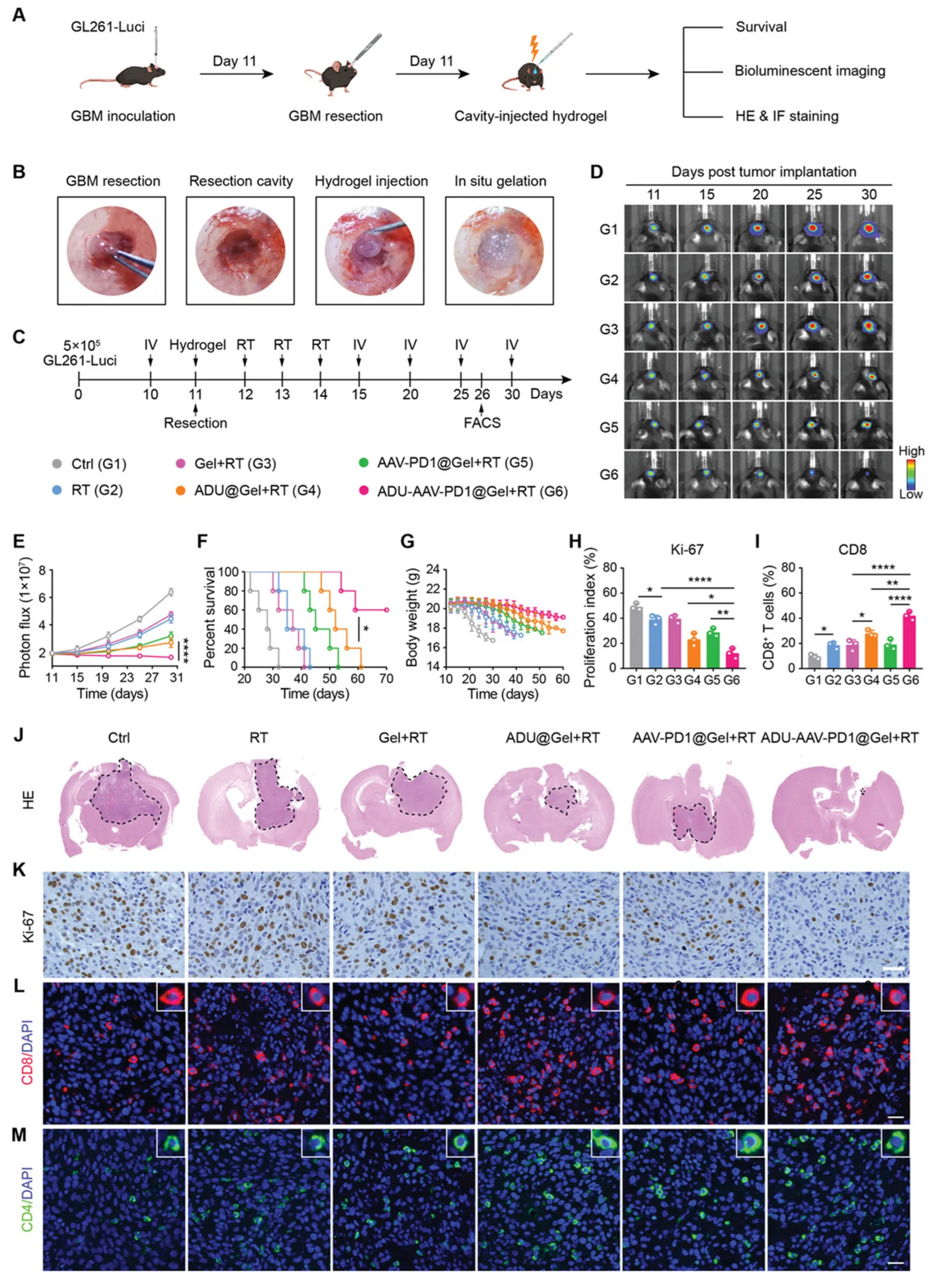
Synergistic radioimmunotherapy using hydrogel suppresses tumor recurrence after GBM resection. (**A**) Schematic workflow for establishing and treating the GBM resection mouse model. (**B**) Intraoperative tumor resection and hydrogel treatment. (**C**) Schematic of the therapeutic evaluation. (**D**) Representative in vivo bioluminescence images and (**E**) quantitative analysis of tumor progression at the indicated time points. (**F**) Kaplan–Meier survival curves and (**G**) body-weight changes in mice in different treatment groups. (**H**) Ki-67 proliferation index and (**I**) quantitative analysis of CD8^+^ T-cell infiltration. (**J**) H&E and (**K**) Ki-67 staining of tumor tissues. (**L**,**M**) Immunofluorescence staining of CD8^+^ and CD4^+^ T cells; DAPI (blue), CD8 (red), and CD4 (green). Scale bars, 50 µm. Data are presented as mean ± SEM. Statistical significance was determined by one-way ANOVA with Tukey’s post hoc test, and survival was analyzed using the log-rank (Mantel–Cox) test. * *p* < 0.05, ** *p* < 0.01, and **** *p* < 0.0001. Rep. from [151], licensed under CC BY-NC-ND 4.0. © [2022].

Local immunochemotherapy has also been used to overcome immune evasion in residual GBM. Because CD47 overexpression suppresses macrophage phagocytosis through SIRPα signaling, a thermosensitive hydroxypropyl chitin (HPCH) hydrogel was engineered to co-deliver α-CD47 and TMZ directly into the resection cavity [150]. The system improved local retention, enhanced macrophage-mediated tumor clearance, recruited CD8^+^ T cells and NK cells, and also effectively bypassed BBB limitations associated with systemic therapy. Similarly, an ROS-degradable hydrogel releasing the STING agonist ADU-S100 and soluble PD-1, when combined with radiotherapy, promoted sustained T-cell infiltration, restored effector function, and generated immune memory against recurrence (Figure 3) [151]. These examples show how degradability and localized release can be used to synchronize chemotherapy, checkpoint modulation, and innate immune activation.

For systemic GBM therapy, nanoscale surface engineering is critical for BBB penetration and tumor homing. A microglia membrane-coated nanoplatform encapsulating a near-infrared II photosensitizer used biomimetic membrane properties to improve BBB crossing and glioma targeting [152]. After 808 nm irradiation, the system induced photodynamic mitochondrial damage and immunogenic cell death, while reprogramming tumor-associated microglia toward a pro-inflammatory M1 phenotype. Similarly, an Angiopep-2-modified LNP improved brain delivery of siRNA, achieving ~2.23% injected-dose brain accumulation [153]. Delivery of PLK1-targeted siRNA suppressed GBM progression and increased median survival by 2.18-fold, demonstrating how peptide modification, lipid composition, and RNA encapsulation can enable brain-targeted gene therapy.

Bioactive ligand-drug systems further show how receptor affinity and stimulus-responsive chemistry can be integrated. A flagellin-based platform targeted Toll-like receptor 5 (TLR5), which is highly expressed on GBM cells, with a monomeric flagellin derivative showing strong receptor affinity [154]. Incorporation of pH-sensitive catechol chemistry enabled conjugation and tumor-acidic release of the boron-containing anticancer drug ixazomib. In 3D GBM spheroids, the ligand-drug conjugate reduced spheroid viability by ~70% and inhibited 20S proteasome activity by ~80%, indicating that immune stimulation and targeted chemotherapy can be combined within one bioactive material design. Overall, GBM biomaterials demonstrate how structure and properties must be combined to overcome disease barriers.

### 4.3. Colorectal Cancer

Colorectal cancer (CRC) is associated with recurrence, metastatic spread, therapeutic resistance, and limited chemotherapy selectivity. Functional biomaterials for CRC are therefore designed to improve drug bioavailability, local retention, immune activation, and pathway-specific targeting by controlling carrier structure, release behavior, and biological responsiveness. At the nanoscale, carrier composition and drug-loading architecture can improve combination therapy. In a study by Zhang D. et al., polymeric NPs co-encapsulating curcumin (CUR) and methotrexate (MTX) were shown to produce sustained, harmonized drug release without an initial burst, improving cellular uptake and apoptosis in CL40 and SW1417 CRC cells compared with free drugs [155]. Similarly, Zhang Q. et al. used an intracellular N-myristoylated self-assembling D-peptide (Gbb-NBD) to localize to the endoplasmic reticulum, Golgi apparatus, and mitochondria, where it disrupted lipid metabolism and induced ferroptotic cell death [156]. This supramolecular design achieved potent activity with a GI_50_ of 500 nM, spared neuronal cells, and avoided acquired resistance, highlighting how intracellular self-assembly can target mechanisms beyond conventional chemotherapy.

Postoperative recurrence emphasizes the importance of local architecture and externally triggered release. For example, Shimizu and Takeoka developed a photothermally responsive multilayer nanosheet device composed of polydopamine-modified polymer films and drug-loaded poly(lactic acid) nanosheets that enabled reversible NIR-triggered chemotherapy release while generating local hyperthermia [157]. Each NIR cycle released a small fraction of the payload, supporting repeated, minimally invasive postoperative treatment. A more immunologically active strategy applied by Liu et al. used a hyaluronic acid/alginate scaffold loaded with oxaliplatin and a biomimetic shPvt1-CM-D nanosystem to silence the oncogenic lncRNA Pvt1 [158]. By combining local drug retention, gene regulation, immunogenic cell death, dendritic-cell activation, and suppression of granulocytic myeloid-derived suppressor cells, the scaffold reduced postoperative local recurrence by 97.8%, decreased distant metastasis by 70.8%, achieved 62.5% survival in perioperative models, and completely prevented tumor rechallenge and metachronous metastasis, resulting in 100% survival.

**Figure 4:**
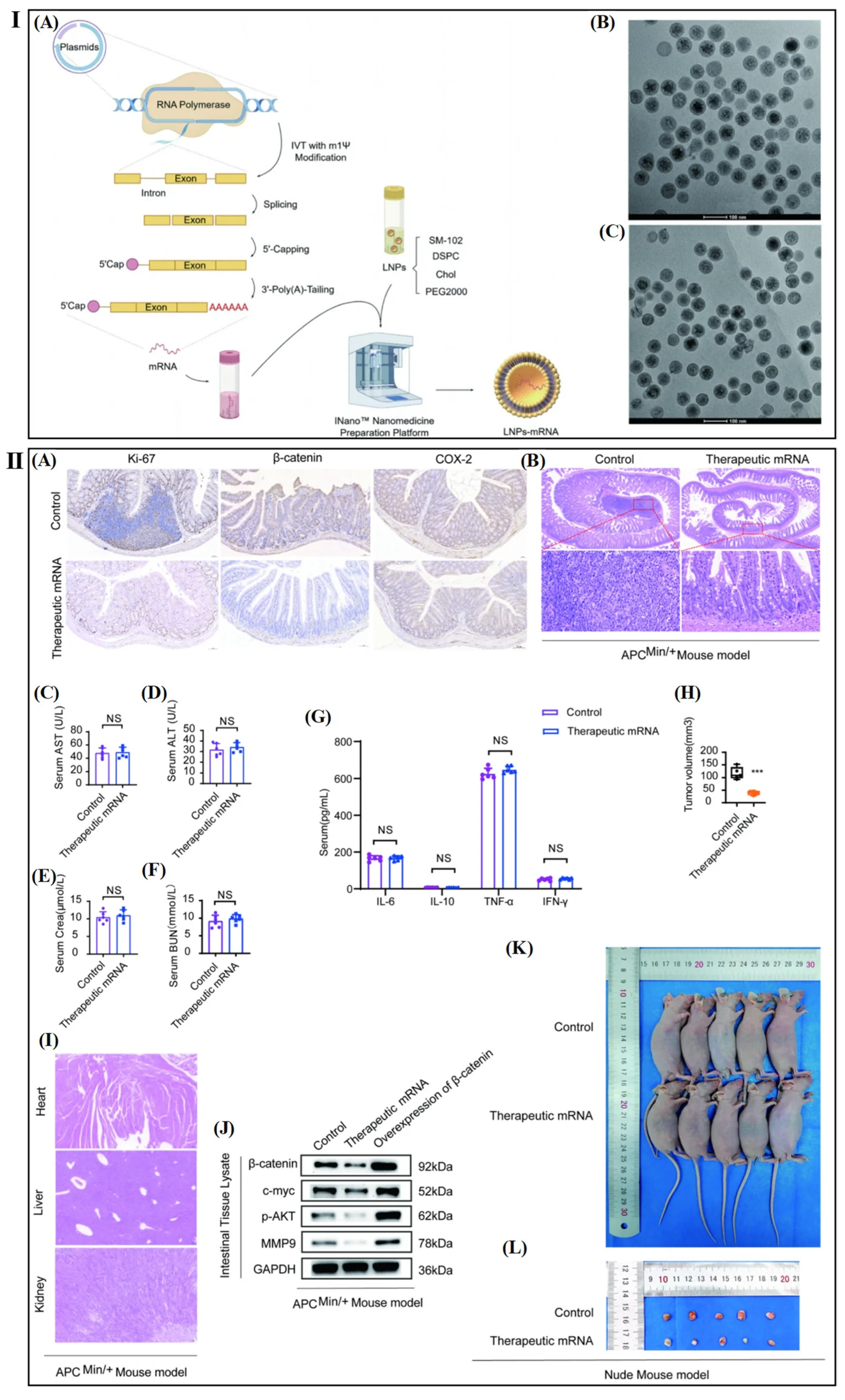
Therapeutic mRNA/LNP platform for colorectal cancer treatment. (**I**) mRNA/LNP synthesis and characterization: (A) Schematic of therapeutic mRNA production from a plasmid template by in vitro transcription, including DNA strand separation, RNA polymerase-mediated transcription, 5′ capping, 3′ polyadenylation, N1-methylpseudouridine incorporation, intron removal, and mRNA generation. Created with FigDraw (https://www.figdraw.com/); (B,C) Cryo-TEM images of blank and mRNA-loaded LNPs, showing well-dispersed spherical particles with no obvious morphological change after mRNA encapsulation. Scale bar = 100 nm. (**II**) In vivo efficiency, mechanism, and safety evaluation: (A) Immunohistochemistry showing reduced Ki-67 and β-catenin expression after therapeutic mRNA treatment, with no significant change in COX-2, indicating suppression of cellular proliferation without marked effects on inflammatory marker expression; (B) H&E staining of intestinal tissues demonstrated preservation of normal mucosal, submucosal, muscular, and serosal architecture in treated APC^Min/+^ mice compared with adenomatous lesions present in controls; (C–F) Serum AST, ALT, creatinine, and BUN levels showing no significant biochemical toxicity; (G) Serum cytokines similarly showing no marked systemic inflammatory response; (H,K,L) Tumor-burden assessment at 8 weeks showing significantly smaller tumors in therapeutic mRNA-treated mice compared with controls (*p* < 0.05; *n* = 5), confirming potent in vivo antitumor activity; (I) H&E staining of heart, liver, and kidney showing no obvious pathological damage; (J) Western blot analysis showing reduced p-AKT and MMP9 expression following mRNA treatment, consistent with inhibition of β-catenin translocation and c-Myc signaling, thereby suppressing MMP9 tumor progression. Data are mean ± SD (*n* = 5); NS means no statistically significant difference between the compared groups; *** *p* < 0.001. Rep. from [159], licensed under CC BY-NC-ND 4.0. © [2026].

Gene-restoration functional biomaterials also offer an approach to treat mutation-driven CRC. In a familial adenomatous polyposis study by Shi et al., APC loss drives adenoma formation, but the large APC protein is difficult to replace with conventional drugs. An mRNA-loaded LNP platform was therefore developed to deliver APC-derived therapeutic peptides after intraperitoneal administration in APC^Min/+^ mice (Figure 4I) [159]. The formulation suppressed adenoma formation and restored tumor-suppressive signaling, including nuclear exclusion of β-catenin, downregulation of c-Myc, AKT1, and MMP9, and recovery of ZO-1 membrane localization (Figure 4II). Safety analyses showed no significant changes in AST, ALT, creatinine, BUN, or inflammatory cytokines, supporting the therapeutic potential of LNP-enabled mRNA delivery. Overall, CRC functional biomaterials show how material structure and properties can be designed to match disease barriers. These examples demonstrate that therapeutic performance depends on integrating architecture, release kinetics, and molecular targeting rather than simply increasing drug dose.

### 4.4. Pancreatic Cancer

Pancreatic cancer remains difficult to treat because its dense desmoplastic stroma, poor vascular perfusion, drug resistance, immune suppression, and early metastasis restrict effective drug delivery. The collagen-rich extracellular matrix (ECM) acts as a major physical barrier by limiting NP transport and chemotherapeutic penetration. Functional biomaterials for pancreatic cancer are therefore designed to adapt their size, respond to tumor stimuli, remodel stromal barriers, and combine therapy with imaging or immune modulation.

Size-adaptive nanocarriers illustrate how structural transformation can improve transport through poorly perfused tumors. For example, Ray et al. engineered hyperbranched polymer-derived nanocarriers to treat acidic tumor conditions by transforming from 150 to 190 nm aggregates into ultrasmall 3–5 nm particles [160]. This pH-triggered size reduction improved diffusion into pancreatic cancer spheroids, enhanced tumor accumulation, and enabled sustained gemcitabine delivery while maintaining compatibility with non-cancerous cells. This design shows how dynamic particle architecture can balance circulation-scale accumulation with deep intratumoral penetration.

Multifunctional local biomaterials have also been developed to integrate imaging, radiotherapy guidance, and therapeutic activity. In a study by Moreau et al., a liquid immunogenic fiducial eluter (LIFE) biomaterial provided MRI and CT contrast for up to three weeks while functioning as a localized immunostimulatory platform [161]. This combined imaging–therapy design significantly suppressed tumor growth and prolonged survival (*p* < 0.0001), demonstrating how material visibility, local retention, and immune activation can be integrated into one pancreatic cancer platform.

**Figure 5:**
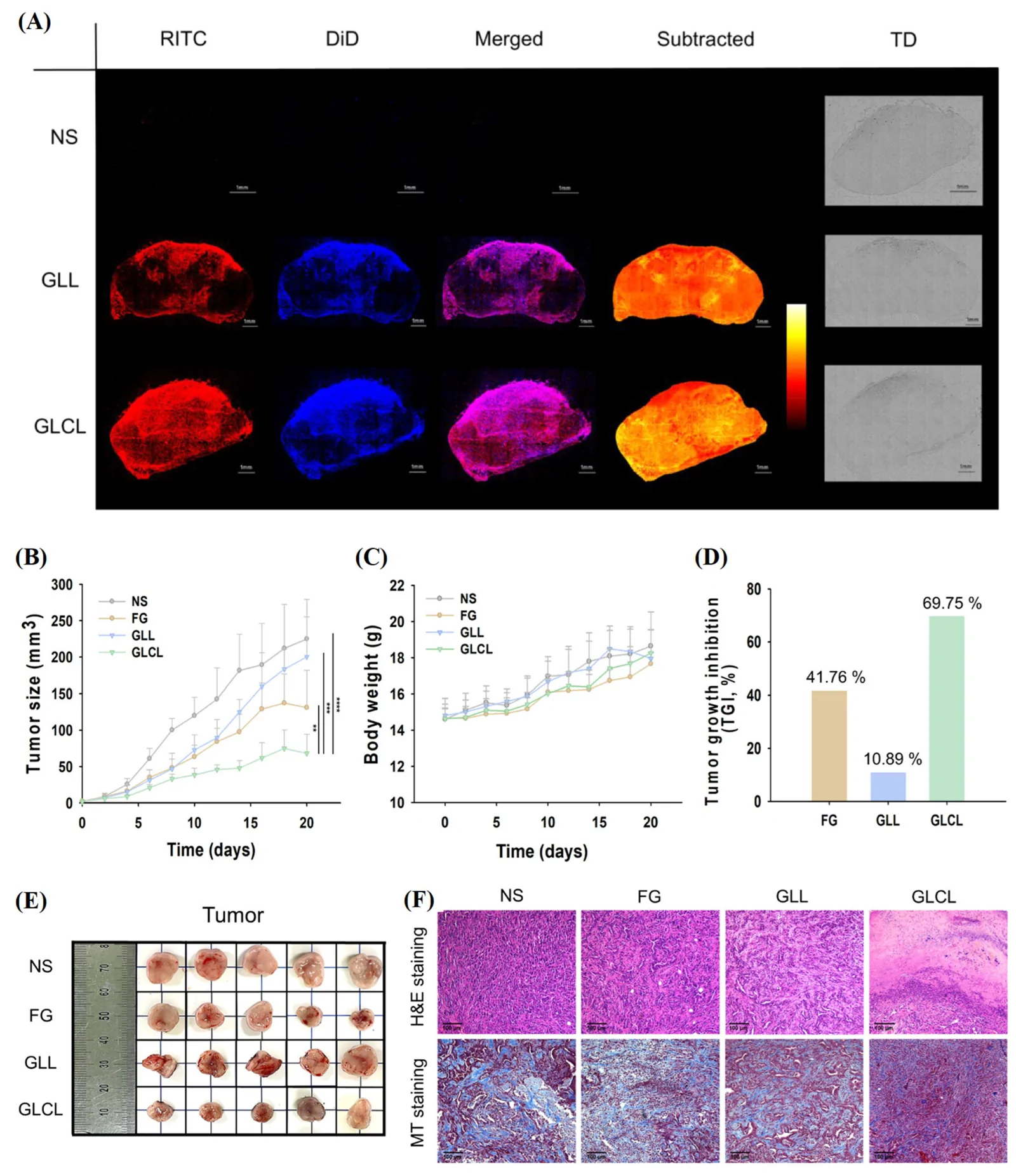
Intratumoral distribution and therapeutic efficiency of gemcitabine-loaded liposomes in KPCY 7160c5 pancreatic tumor-bearing mice: (**A**) Confocal images of centrally sectioned tumors from mice treated with normal saline (NS), gemcitabine-loaded liposomes (GLL), or gemcitabine-loaded collagenase-functionalized liposomes (GLCL). RITC was encapsulated within the hydrophilic core to track gemcitabine distribution, whereas DiD was incorporated into the lipid bilayer to visualize liposome localization. RITC (red) indicates gemcitabine distribution, DiD (blue) indicates liposome localization, the merged images show channel overlap in magenta/purple. The subtracted images display the normalized RITC–DiD signal using a pseudocolor scale, with lighter yellow/white tones indicating higher signal intensity and darker red/black tones indicating lower intensity. TD is indicates transmitted light. Scale bar = 1 mm; (**B**–**D**) Therapeutic evaluation after treatment with NS, free gemcitabine (FG), GLL, or GLCL, showing tumor volume, body weight, and tumor growth inhibition. Data are mean ± SD; significance was determined by one-way ANOVA with Tukey’s post hoc test (** *p* < 0.01, *** *p* < 0.001, **** *p* < 0.0001). (**E**) Representative excised tumors collected at the study endpoint. (**F**) Histopathological analysis of tumor sections stained with hematoxylin and eosin (H&E; upper row) and Masson’s trichrome (MT; lower row). Scale bar = 100 µm. Rep. from [162], licensed under CC BY-NC-ND 4.0. © [2026].

Stromal remodeling is another important strategy for improving drug penetration. Gemcitabine-loaded collagenase-conjugated liposomes (GLCLs) were designed by Hwang et al. to enzymatically loosen collagen barriers and promote more homogeneous intratumoral drug distribution [162]. This architecture achieved 69.8% tumor-growth inhibition, nearly sixfold higher than conventional gemcitabine liposomes at the same drug dose (10.9%). Multiscale imaging further confirmed enhanced molecular-level drug penetration, linking ECM-modifying surface functionality directly to improved therapeutic performance (Figure 5).

Stimuli-responsive nanocarriers can also address both physical and immunological barriers. Hypoxia- and acidity-responsive NPs co-loaded with the gemcitabine prodrug GemC18 and the TGF-β/SMAD inhibitor galunisertib were designed to trigger transcytosis, undergo tumor-associated charge reversal, inhibit stromal proliferation, and reduce immune suppression [163]. In this system, Zhang et al. used gemcitabine to provide direct cytotoxicity, while galunisertib remodeled the matrix and supported antitumor immunity. Overall, pancreatic cancer biomaterials show that therapeutic performance depends on engineering size adaptability, stromal penetration, stimulus-responsive release, imaging capability, and immune remodeling to overcome the restrictive pancreatic tumor microenvironment.

Beyond ECM remodeling alone, recent pancreatic cancer biomaterials increasingly target both tumor cells and stromal compartments to improve penetration, suppress metastasis, and reverse immune dysfunction. In a study by Zhu et al., ATF peptide-decorated liposomes were co-loaded with cisplatin and rapamycin (ATF@Pt/Rapa Lps) and used uPAR-mediated targeting to act on both pancreatic cancer cells and cancer-associated fibroblasts [164]. This dual-targeting design enhanced tumor penetration and disrupted stromal architecture by downregulating α-SMA, collagen I, and fibronectin, leading to stronger inhibition of tumor proliferation, migration, and liver metastasis than non-targeted formulations. In a similar evaluation by Chen et al., aptamer-decorated hypoxia-responsive NPs co-delivering gemcitabine monophosphate and the STAT3 inhibitor HJC0152 were engineered to undergo hypoxia-triggered size reduction and charge reversal, producing ultrasmall particles for deeper tumor penetration [165]. The gemcitabine provided cytotoxic activity, while HJC0152 suppressed aberrant STAT3 signaling in tumor and stromal cells, softened the stromal barrier, and shifted the tumor microenvironment toward immune activation. Together, these studies show that pancreatic cancer biomaterials are progressing from simple drug carriers toward adaptive, multifunctional systems that integrate stromal remodeling, penetration enhancement, immune modulation, and metastasis suppression.

### 4.5. Melanoma

Melanoma is highly metastatic and often limited by poor tumor selectivity, systemic toxicity, and an immunosuppressive tumor microenvironment (TME). Functional biomaterials for melanoma are therefore being designed to improve tumor-specific activation, targeted uptake, immune remodeling, and postoperative tissue repair by controlling carrier chemistry, surface functionality, stimulus responsiveness, and local retention.

**Figure 6:**
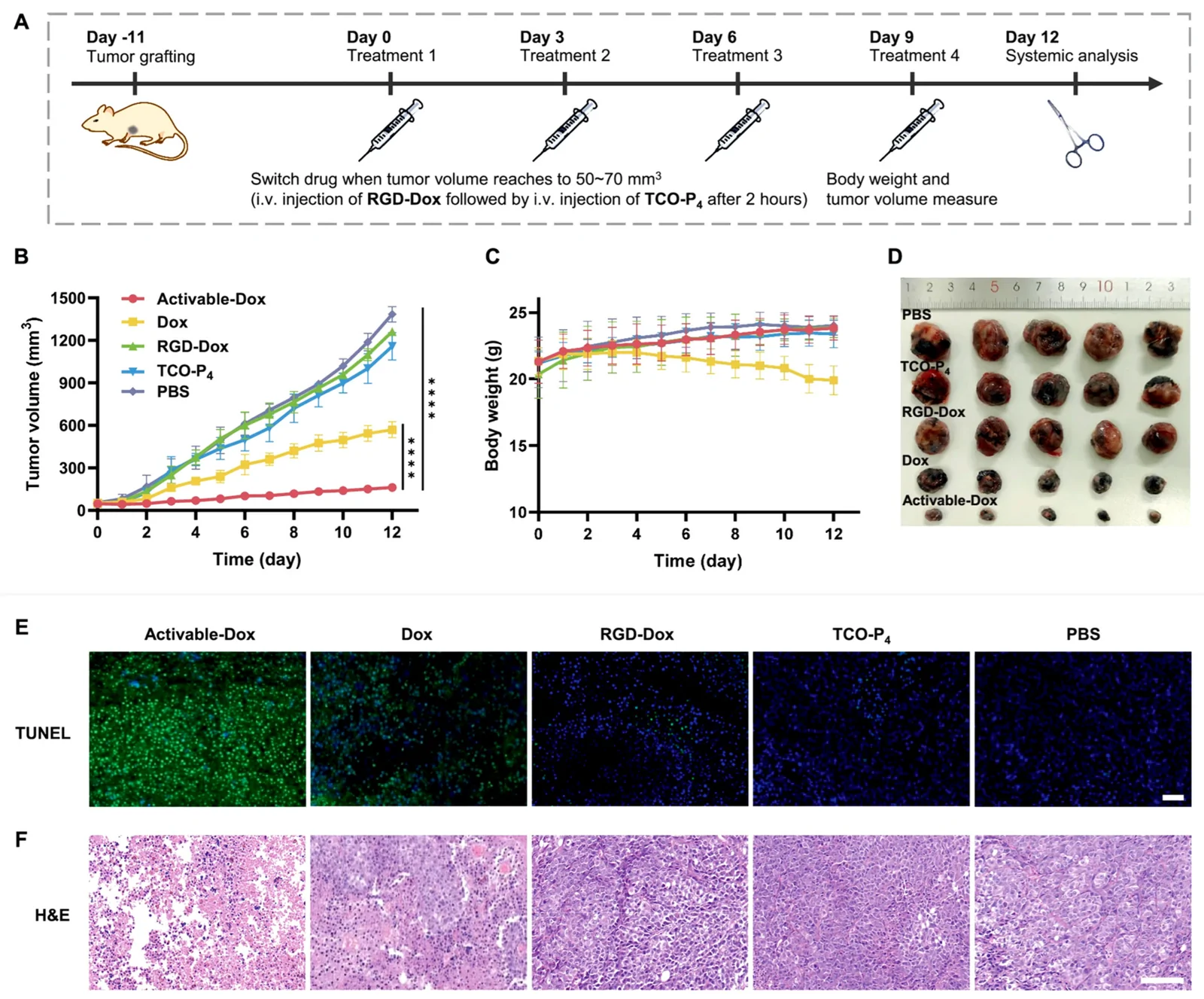
Evaluation of therapeutic efficiency in the melanoma tumor-bearing mouse model. (**A**) Schematics of the treatment regimen, showing four treatment administrations with daily monitoring of tumor volume and body weight. (**B**) Tumor growth profiles throughout the treatment period (*n* = 5 mice per group). Tumor volume was calculated using the formula V = 0.5 × (minor axis) × (major axis)^2^. Statistical comparisons were performed by one-way ANOVA followed by Tukey’s post hoc test (**** *p* < 0.0001). (**C**) Body-weight changes recorded during the study (*n* = 5 mice per group). (**D**) Representative photographs of excised tumors were collected 12 days after treatment initiation. (**E**,**F**) Histopathological analysis of harvested tumor tissues by TUNEL staining and H&E staining, respectively, demonstrates treatment-induced cellular and tissue alterations. Scale bar = 100 µm. Data are expressed as mean ± SD. Rep. from [166], under the Creative Commons Attribution License (CC BY).

Bioorthogonal chemistry provides one approach to improve drug activation specificity. In a systematic study, He et al. developed a 3-vinyl-6-oxymethyl-tetrazine (voTz)-based platform that enabled modular construction of tetrazine-caged prodrugs and peptide–prodrug conjugates capable of activation [166]. Unlike endogenous stimulus-responsive systems that may activate prematurely, these prodrugs remained stable in circulation, selectively bound target cells, and underwent controlled intracellular activation, thereby improving antitumor potency while reducing off-target toxicity (Figure 6). In another study by Imumkachi et al., tryptophan-functionalized carbon dots, especially tri-Trp-CDs, were exploited to target LAT1 overexpression in SK-MEL-2 melanoma cells to enhance doxorubicin uptake and cytotoxicity while improving selectivity over normal kidney cells [167]. These examples show how chemical caging and transporter-targeted surface design can expand the therapeutic performance of cytotoxic drugs.

Immunomodulatory nanocarriers further illustrate how material composition can convert local tumor inhibition into systemic immune activation. Hyaluronic acid-based HTCS NPs were designed by Liao et al. to co-deliver the STING agonist 2′3′-cGAMP and the mitochondria-targeting photosensitizer TPP-PEI-Ce6 [168]. It was reported that tumor uptake enabled both mitochondrial ROS generation and photodynamic therapy, while immunogenic cell death and STING activation promoted dendritic-cell maturation, inflammatory cytokine secretion, and infiltration of cytotoxic T cells and NK cells. PLGA NPs co-loaded with doxorubicin, losartan, and metformin were also engineered by Ramzy et al. to act on melanoma cells, tumor-associated fibroblasts, and tumor-associated macrophages [169]. However, while dual-drug combinations improved outcomes, the triple-drug formulation promoted immune depletion and metastasis, emphasizing that multifunctional biomaterials require optimized drug ratios rather than maximal payload complexity.

Melanoma biomaterials are also being extended to postoperative therapy and tissue regeneration. Liu et al. fabricated a collagen-derived bioelectronic skin scaffold (c-ADM) by incorporating PEDOT and copper sulfide NPs into porcine acellular dermal matrix, combined with photothermal therapy, electrostimulation, and controlled doxorubicin release [170]. Under NIR irradiation, CuS NPs produced local hyperthermia with ΔT > 40 °C, inducing melanoma-cell apoptosis and sustained drug release. Its pH-responsive Cu^2+^ release further enabled antibacterial activity under acidic tumor-associated conditions (pH 5.0–6.0) and supported tissue repair under physiological conditions (pH 7.4), linking scaffold architecture and responsive chemistry to both tumor control and wound management.

Beyond biochemical targeting, ionic remodeling is emerging as a functional biomaterial strategy. Because extracellular K^+^ accumulation suppresses CD8^+^ T-cell metabolism, proliferation, and effector function, sodium zirconium cyclosilicate (ZS-9) was encapsulated in a thermosensitive PLGA-PEG-PLGA hydrogel to create a localized K^+^-scavenging depot [171]. This ionic-regulating hydrogel restored T-cell metabolic fitness, reduced exhaustion, and enhanced adoptive cell therapy. Overall, melanoma biomaterials demonstrate how therapeutic performance can be improved by integrating bioorthogonal activation, transporter-mediated uptake, immune stimulation, photothermal/electrical functionality, controlled release, and ionic TME remodeling.

### 4.6. Bladder and Ovarian Cancers

Bladder and ovarian cancers present different therapeutic barriers, including surgical complications, multidrug resistance (MDR), and poor tumor-specific delivery. Functional biomaterials in these settings are being used not only as drug carriers but also as structural reinforcements, gene-silencing platforms, and theranostic systems in which material architecture and surface chemistry directly influence clinical or experimental performance. In bladder cancer, Krughoff et al. evaluated remodelable bovine pericardial bolsters as mechanical buttresses during radical cystectomy and urinary diversion [172]. Among 398 patients, enterocutaneous fistula occurred in 7 of 301 non-buttressed cases (2.3%) but was not observed in the 97 buttressed cases. Although this reduction was not statistically significant (OR = 0.20; 95% CI: 0.01–3.56; *p* = 0.20), biomaterial reinforcement significantly reduced postoperative functional bowel obstruction (OR = 0.28; 95% CI: 0.08–0.93; *p* = 0.03). Although the primary endpoint did not reach statistical significance, this study provides a clinically relevant example of how biomaterial-based structural reinforcement may support selected perioperative outcomes in complex oncologic surgery.

To strengthen local bladder drug exposure, reverse thermal hydrogel systems have also been developed for intravesical chemotherapy. UGN-102, a mitomycin-containing reverse thermal hydrogel, was administered as a cooled liquid that converts into a semi-solid gel depot at body temperature after bladder instillation. This formulation was designed to prolong mitomycin dwell time and contact with the bladder surface, thereby improving local chemoablation compared with rapidly diluted aqueous drug preparations. In the OPTIMA II phase 2b study by Chevli et al. of patients with low-grade intermediate-risk non-muscle-invasive bladder cancer, six once-weekly intravesical instillations of UGN-102 produced a 65% complete response rate at 3 months, with 61% of complete responders remaining disease-free at 12 months after treatment initiation. This example directly links biomaterial phase-transition behavior, local retention, and therapeutic performance in bladder cancer [173].

**Figure 7:**
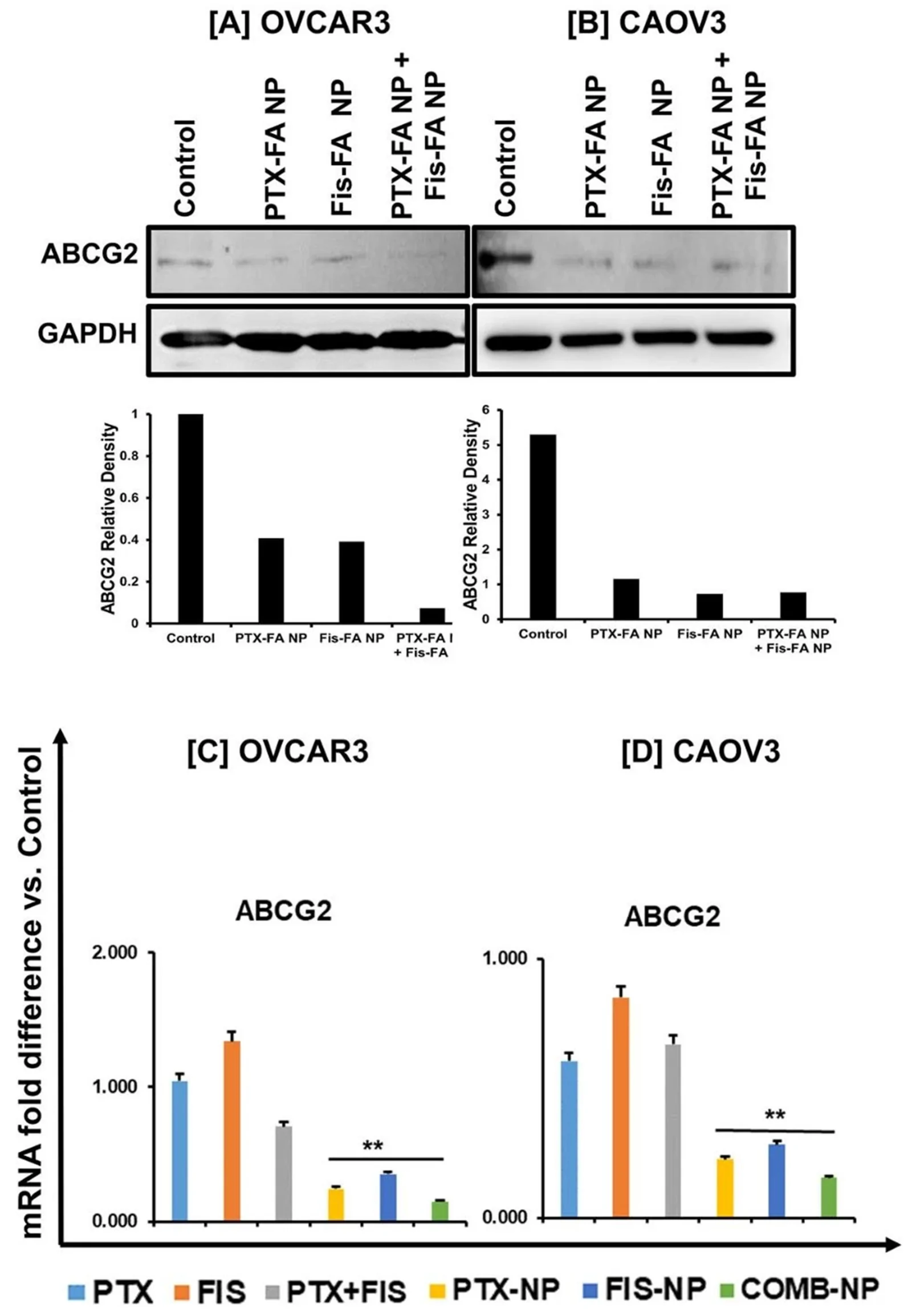
PBM NPs downregulate the MDR gene ABCG2 in OvCa cells. ABCG2 protein expression in (**A**) OVCAR3 and (**B**) CAOV3 cells following 48 h treatment with Fis-FA or PTX-FA NPs, alone or in combination, assessed by Western blotting with GAPDH as the loading control; densitometric analysis is shown below. ABCG2 mRNA expression in (**C**) OVCAR3 and (**D**) CAOV3 cells was quantified by RT-PCR and normalized to 18S rRNA, with fold changes calculated relative to untreated controls. Data are presented as mean ± SEM; ** *p* < 0.01 (Student’s *t*-test). Rep. from [175], under the Creative Commons Attribution License (CC BY).

In ovarian cancer, Yang et al. used hyaluronic acid-based self-assembling NPs composed of HA-PEI/HA-PEG to overcome MDR through CD44-mediated targeting [174]. The HA surface chemistry enabled selective interaction with CD44-overexpressing paclitaxel-resistant OVCAR8TR cells, while the PEI component supported MDR1 siRNA delivery. This design downregulated MDR1 and P-glycoprotein expression, reduced efflux activity, and restored paclitaxel sensitivity, leading to marked tumor-growth inhibition in MDR ovarian cancer xenografts. In the same vein, paclitaxel conjugated with folic acid (PTX-FA) and fisetin conjugated with folic acid (Fis-FA) planetary-ball-milled (PBM) NPs targeted FRα-overexpressing OvCa cells. The developed system reduced the required PTX concentration fivefold, enhanced apoptosis from 14.4% to 80.4% in OVCAR3 and 2.69% to 90.0% in CAOV3, increased BAK and active caspase-3, and reversed ABCG2-mediated MDR (Figure 7) [175]. These studies demonstrate how receptor-targeted surface chemistry and RNA-interference delivery can reverse chemoresistance rather than simply increase chemotherapy dose.

Luong et al. developed a folate-targeted theranostic nanoplatform composed of a superparamagnetic iron oxide NP (SPION) core coated with folic acid-conjugated PAMAM dendrimers and loaded with 3,4-difluorobenzylidene-curcumin (CDF) [176]. The SPION core provided magnetic resonance imaging capability, while the folate-functionalized dendrimer shell enhanced uptake in folate receptor-positive SKOV3 and HeLa cells. Compared with non-targeted formulations, SPIONs@FA-PAMAM-CDF produced stronger anticancer activity, increased apoptosis through PTEN and caspase-3 upregulation, and suppressed NF-κB signaling. This illustrates how core–shell architecture, ligand functionalization, and imaging-active composition can be integrated to achieve simultaneous tumor targeting, therapy, and diagnosis.

Overall, bladder and ovarian cancer examples show that biomaterial performance depends on matching structure and properties to the clinical barrier being addressed. These systems highlight the broader role of functional biomaterials in improving surgical safety, overcoming chemoresistance, and enabling precision theranostics.

### 4.7. Bone Cancer and Metastasis

Bone cancer therapy must address two critical clinical challenges, including eliminating residual tumor cells after resection and regenerating the resulting bone defect. Functional biomaterials for this application are therefore increasingly designed as theragenerative systems, where composition, architecture, degradation behavior, and stimulus responsiveness are engineered to support both tumor ablation and bone repair.

Bioactive glass-based nanomaterials illustrate how inorganic composition and surface modification can integrate imaging, therapy, and regeneration. Chen et al. developed fetal bovine serum-decorated europium-doped bioactive glass NPs (EuBGN@FBS) for tumor-specific therapy and bioimaging [177]. Europium doping provided imaging functionality, while FBS surface decoration improved dispersibility, hemocompatibility, cellular uptake, and tumor-targeting efficiency. The system also enabled pH-responsive drug release and enhanced in vivo imaging with negligible side effects, demonstrating how ion doping and protein-based surface engineering can improve both diagnostic and therapeutic performance. Similarly, Shoaib et al. reported magnesium-doped mesoporous bioactive glass NPs (Mg-MBG NPs) with a nanoscale diameter of 65 ± 5 nm for mitomycin C delivery and apatite formation [178]. The mesoporous architecture supported drug loading and achieved up to 89% cumulative release under acidic conditions (pH 6.4), while drug-loaded Mg-MBG suppressed MG-63 osteosarcoma cells with an IC_50_ of 20.8 µg/mL. At the same time, the material maintained cytocompatibility and promoted hydroxycarbonate apatite formation, linking mesoporosity and Mg doping to both anticancer and osteogenic functions.

**Figure 8:**
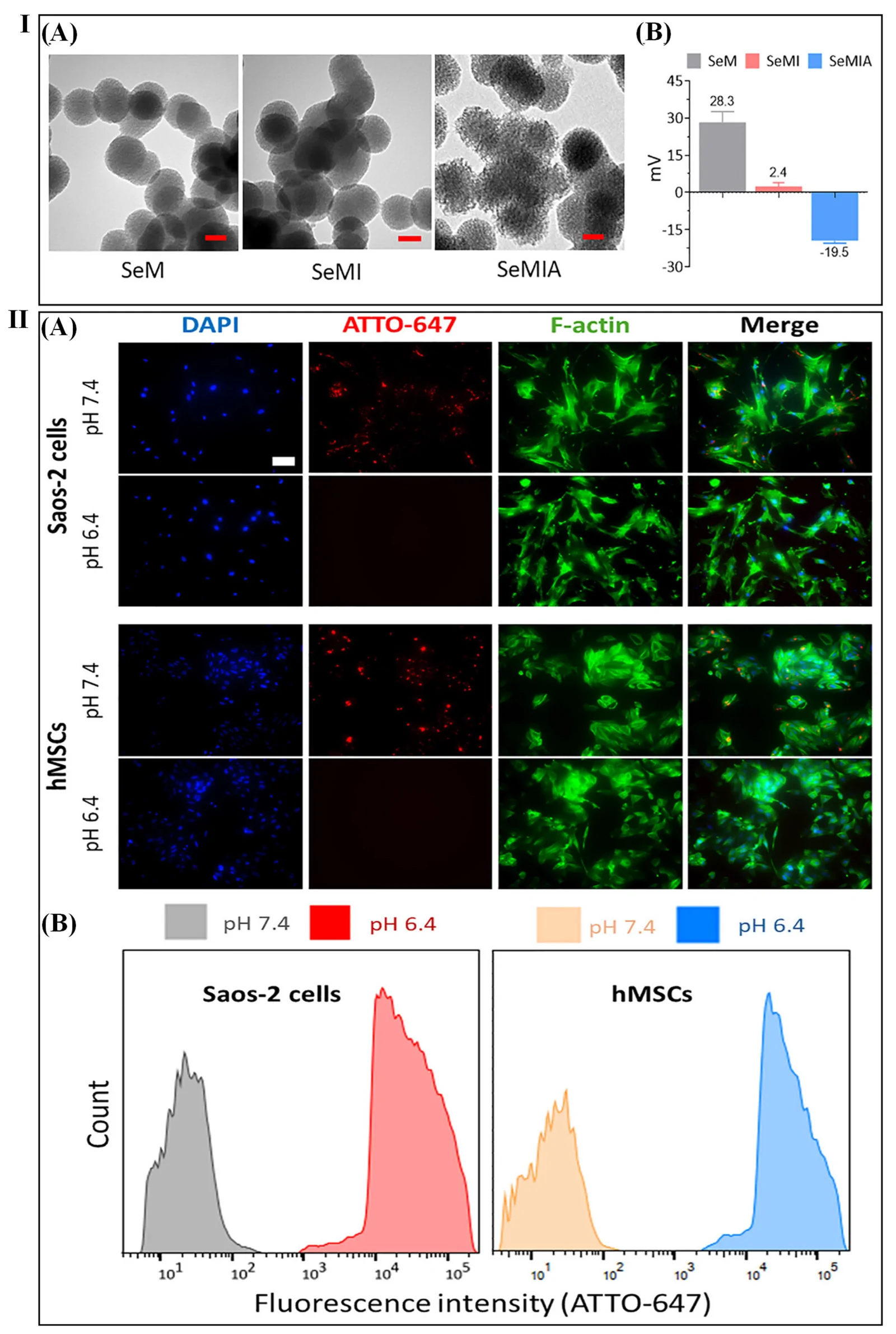
Physicochemical characterization and bone cancer cellular internalization of functionalized selenium-doped mesoporous silica NPs (SeMSNs) for bone cancer therapy. (**I**). Physicochemical characterization of ALN/imine-functionalized selenium-doped mesoporous silica NPs (SeMIA). (A) TEM micrographs of Se-doped MSNs (SeM), imine-modified SeM (SeMI), and ALN/imine-functionalized SeM (SeMIA), illustrating morphological features and particle architecture (scale bar = 50 nm). (B) Zeta potential measurements of SeM, SeMI, and SeMIA determined by DLS in neutral high-purity water, demonstrating surface charge modifications following sequential functionalization. (**II**). Cellular uptake evaluation of 647MIA@TCP granules. (A) Fluorescence microscopy images of Saos-2 cells and human mesenchymal stem cells (hMSCs) following incubation with two 647MIA@TCP granules (647MIA@TCP2) under physiological (pH 7.4) and acidic (pH 6.4) conditions for 24 h. F-actin was stained with phalloidin (green), nuclei with DAPI (blue), and NP internalization was assessed by fluorescence signal distribution (scale bar = 200 µm). (B) Quantitative flow cytometric analysis of NP uptake by Saos-2 cells and hMSCs after 24 h exposure to 647MIA@TCP2 at pH 7.4 and pH 6.4. ATTO-647-labeled NPs are shown by the red fluorescence signal. Rep. from [179], licensed under CC BY-NC-ND 4.0. © [2026].

He et al. further advanced pH-responsive bone cancer biomaterials by functionalizing β-tricalcium phosphate (β-TCP) granules with selenium-doped mesoporous silica NPs (SeMSNs) through tumor-sensitive imine bonds [179]. The resulting SeMIA@TCP system was designed to remain stable at physiological pH but release NPs under acidic tumor-like conditions through imine bond cleavage. This pH-triggered release enhanced osteosarcoma-selective NP internalization while supporting osteogenic differentiation and mineralization of human mesenchymal stem cells (Figure 8). The system demonstrates how calcium phosphate architecture, selenium doping, and acid-cleavable surface chemistry can be combined to achieve tumor selectivity and bone-regenerative activity.

Stimuli-responsive hydrogels and scaffolds have also been used to combine localized tumor ablation with bone regeneration. Chen et al. developed dual-crosslinked alginate/gelatin hydrogels loaded with doxorubicin and polydopamine (ALG/GelAGE-PDA@DOX) for chemo-photothermal therapy [180]. The hydrogel network enabled controlled drug release, while polydopamine provided photothermal heating to eliminate MG63 osteosarcoma cells. Sustained strontium ion release further increased alkaline phosphatase activity and promoted osteogenic differentiation, demonstrating how crosslinking chemistry, photothermal components, and ion release can be coordinated within one platform. Pektas et al. similarly incorporated MXene into 3D-printed silk fibroin aerogel scaffolds to integrate photothermal tumor ablation with bone regeneration [181]. Under NIR irradiation, the scaffold reached 45–53 °C, enabling efficient osteosarcoma ablation while supporting preosteoblast proliferation, mineral deposition, and local drug delivery.

Beyond photothermal therapy, Huang et al. developed ffBT-T NPs as a sonodynamic platform for osteosarcoma treatment and bone repair [182]. Ultrasound activation generated reactive oxygen species that induced apoptosis, ferroptosis, and immunogenic cell death in osteosarcoma cells. Importantly, the same platform activated PI3K-Akt signaling and enhanced osteogenic differentiation of bone marrow mesenchymal stem cells by 2.1-fold. In vivo, this dual-functional design suppressed tumor progression while accelerating bone regeneration, showing how acoustic responsiveness can expand postoperative osteosarcoma therapy beyond heat-based strategies.

Implantable drug-releasing bone substitutes further show how internal architecture controls the balance between mechanical function and local chemotherapy. Wang et al. engineered conventional dense polymethylmethacrylate (PMMA) bone cement into a porous cisplatin-loaded scaffold using carboxymethylcellulose (CMC) as a porogen [183]. Porosity increased cisplatin release to 18% over 28 days, compared with <2% from conventional PMMA, and increased osteosarcoma cell death to 91.3% after 7 days. Importantly, ≤3% CMC preserved clinically acceptable compressive strength, demonstrating the need to balance pore formation, drug release, and load-bearing integrity. In a related strategy, Wang et al. functionalized commercially available Bio-Oss^®^ and MBCP^®^+ bone substitute granules with cisplatin [184]. These granules produced dose-dependent cytotoxicity against metastatic breast and prostate cancer cells, had minimal effects on human bone marrow stromal cells, supported bone ingrowth and new bone formation in vivo, and showed negligible systemic cisplatin accumulation. Collectively, bone cancer biomaterials demonstrate how structure and properties regulate therapeutic performance across multiple length scales. These systems reflect the transition from single-purpose implants or drug carriers toward multifunctional theragenerative platforms that integrate tumor eradication, controlled release, imaging, bone regeneration, and reconstruction after tumor resection.

## 5. Future Perspectives

### 5.1. AI-Guided Biomaterial Design

A major future direction in pharmaceutical technology is the integration of artificial intelligence (AI) and machine learning (ML) into drug-delivery system design [185]. This is particularly relevant for functional biomaterials used in localized cancer therapy, where drug release is governed by multiple interacting structure-property variables, such as material composition, architecture, surface chemistry, degradation rate, and tumor-specific stimuli. Because drug release determines the local availability of therapeutic agents, accurate release prediction is essential for optimizing therapeutic efficiency, controlling dosing rate, limiting systemic toxicity, and maintaining drug concentrations [186,187].

Conventional mathematical models remain useful for describing drug-release behavior. Zero-order, first-order, Higuchi, and Hixson–Crowell models are commonly used to describe release kinetics, while Korsmeyer–Peppas and Weibull models help interpret release mechanisms such as diffusion, swelling, erosion, or degradation-controlled transport [188]. However, these models are often limited when applied to modern multifunctional biomaterials because release may result from several coupled processes, including heterogeneous pore diffusion, carrier degradation, environmental responsiveness, multi-drug loading, and changing tumor conditions [189]. Therefore, classical kinetic models may describe observed release trends but may not fully predict complex release behavior during biomaterial design. A recent example illustrates this advantage. Zhang et al. developed DrugNet, a multilayer perceptron neural-network model, to predict in vitro release curves from PLGA-based drug-delivery systems using a literature-derived dataset of 39 release profiles and physicochemical descriptors of PLGA carriers and drug molecules. Compared with Korsmeyer–Peppas and Weibull semi-empirical models, DrugNet reduced the mean squared error by 20.994 and 1.561, respectively, and increased R^2^ by 0.036 and 0.005, respectively. Although not cancer-specific, this example demonstrates how ML can better capture nonlinear formulation–release relationships that are difficult to represent using fixed mathematical release models [190].

AI and ML provide complementary tools for overcoming these limitations by learning nonlinear relationships directly from experimental or computational datasets. In this context, ML can connect biomaterial structure and formulation variables with performance outputs such as burst release, cumulative release, release half-life, diffusion exponent, degradation rate, and formulation stability. Supervised ML methods, including regression models, decision trees, random forests, support vector machines, gradient boosting algorithms, and artificial neural networks, have been used to model formulation–release relationships [189,191]. Simpler models may offer greater interpretability, whereas neural networks and ensemble methods may better capture complex nonlinear interactions when sufficient data are available.

Unsupervised approaches can also support biomaterial design by identifying hidden patterns within formulation and release datasets. For example, clustering methods such as K-means can group formulations with similar release profiles or classify materials according to shared physicochemical features [189,191]. Such analysis can guide formulation screening, reduce experimental trial-and-error, and identify design regions associated with sustained release, minimal burst effect, or stimulus-responsive behavior.

For functional biomaterials in localized cancer therapy, the most promising approach is likely to combine ML with mechanistic understanding. Hybrid models could incorporate experimentally derived parameters, such as diffusion coefficients, degradation constants, or empirical release exponents, as inputs for predictive algorithms. This would allow AI-guided models to remain connected to biomaterial mechanisms while improving prediction accuracy. Future progress will depend on standardized release datasets, consistent testing conditions, and experimental validation of AI-predicted formulations.

### 5.2. Personalized Biomaterial Design

Personalized medicine is based on the recognition that patients differ in tumor genetics, physiology, immune status, treatment history, and environmental exposure, and may therefore require disease interventions tailored to these individual characteristics [192]. For localized cancer therapy, this concept extends beyond selecting the appropriate drug; it also involves designing biomaterials whose structure, properties, and therapeutic functions are matched to the patient’s tumor biology, anatomical site, and treatment objective.

In tissue engineering and drug delivery, biomaterials provide structural and biochemical cues that regulate cell adhesion, proliferation, migration, differentiation, and tissue repair [193]. In a personalized SPP framework, these cues can be adjusted through scaffold geometry, porosity, degradation rate, surface chemistry, and functionalization. For example, patient-specific tumor location, residual cavity geometry, vascularity, receptor expression, and immune profile could guide the selection of injectable hydrogels, implantable depots, or 3D-printed scaffolds for localized cancer treatment.

AI and ML can support this transition by analyzing complex patient and tumor datasets to improve cancer classification, diagnosis, therapeutic selection, and biomaterial optimization [194]. When integrated with mathematical and in silico models, AI/ML can simulate NP transport across systemic, tissue, and cellular scales, helping predict biodistribution, tumor penetration, cellular uptake, and drug-release performance [195]. These tools can also anticipate interactions between biomaterials and biological fluids and cell membranes, thereby enabling more rational selection of carrier size, surface charge, ligand type, degradability, and release profile [194]. Personalized biomaterial design is particularly relevant in cancers with strong patient-to-patient heterogeneity. In neuro-oncology, for example, AI and ML can help predict glioma genotype, phenotype, prognosis, chemotherapy sensitivity, and immunotherapy outcomes [196].

Such predictions could guide the design of patient-specific postoperative depots, BBB-penetrating nanocarriers, immune-modulating hydrogels, or locally implanted release systems. Similarly, deep learning models that integrate imaging, surgical records, medical history, and pathology data may help surgeons estimate recurrence risk, select treatment strategies, and design biomaterial-assisted reconstruction or localized therapy plans [197].

Manufacturing technologies will also be important for clinical translation. The integration of 3D printing, inkjet printing, fused deposition modeling, Internet of Things-enabled healthcare systems, and blockchain-supported prescription workflows may enable decentralized and secure production of tailored drug-delivery systems [198]. In this context, patient-specific implants or depots could be designed to match surgical defects, local mechanical requirements, and desired release duration while improving access to customized medicines.

Despite this potential, personalized biomaterial design requires careful validation before broad clinical adoption. Major barriers include data heterogeneity, limited standardized datasets, regulatory complexity, reproducibility of customized formulations, model interpretability, and the need for external validation across diverse patient populations [196]. Future progress will depend on combining AI-guided prediction with mechanistic biomaterials knowledge, clinically relevant testing models, and scalable manufacturing approaches. Ultimately, personalized biomaterials should be designed not only to deliver drugs, but to match material architecture, biological function, and release behavior to the therapeutic needs of each patient.

### 5.3. Image-Guided Localized Delivery

Image-guided and minimally invasive delivery will be central to the clinical translation of functional biomaterials for localized cancer therapy. AI and ML are increasingly being integrated into minimally invasive and image-guided procedures to improve preoperative planning, intraoperative navigation, and postoperative assessment [199]. For example, in gastrointestinal and laparoscopic surgery, ML approaches, including supervised learning, reinforcement learning, and imitation learning, have been used to support lesion detection, workflow automation, cancer screening, and prediction of surgical outcomes [200,201]. These capabilities are relevant to localized biomaterial delivery because they can help identify the optimal injection or implantation site, avoid critical anatomical structures, and standardize placement of drug-loaded depots or scaffolds.

AI-assisted imaging can also improve delivery precision by enhancing tissue visualization and anatomical segmentation. In intestinal ultrasonography, AI tools can support automated assessment of wall thickness, vascularity, and tissue features such as inflammatory or fibrotic strictures, which are important for patient-specific treatment planning [202]. Similar ML and deep learning approaches in gynecologic laparoscopy can classify and segment anatomical structures, detect surgical instruments, recognize procedural phases, provide real-time decision support, and automate operative documentation [203]. For localized cancer therapy, these tools could guide biomaterial placement in anatomically complex regions and improve procedural reproducibility.

Ultrasound and MRI are particularly relevant for minimally invasive biomaterial delivery because they can provide real-time or high-resolution feedback during treatment. AI-enhanced ultrasound can improve image acquisition, quality assessment, segmentation, classification, and real-time monitoring using models such as CNNs, U-Net, DenseNet, and other neural networks [204]. MRI is also being transformed by AI through noise reduction, super-resolution reconstruction, artifact correction, improved tissue contrast, automated patient positioning, adaptive sampling, and faster image reconstruction [205,206]. These advances could support the development of imaging-visible hydrogels, magnetic or radiopaque NPs, and implantable depots whose distribution, retention, degradation, and therapeutic response can be monitored noninvasively.

Future image-guided biomaterial systems should therefore integrate therapeutic function with imaging compatibility and minimal invasion. When combined with AI-assisted imaging and robotic delivery, these properties could enable real-time monitoring, adaptive dosing, and more accurate evaluation of treatment response. However, clinical adoption will require standardized imaging protocols, validated AI models, regulatory clarity, and demonstration that image-guided placement improves therapeutic outcomes compared with conventional delivery.

### 5.4. Smart Multi-Responsive Biomaterial Systems

The development of biomaterials has traditionally relied on trial-and-error experimentation, extensive testing, and empirical knowledge [207]. However, the field is increasingly shifting toward data-driven design, where AI, ML, and predictive modeling are used to guide material selection, optimize formulation parameters, and improve therapeutic performance [208]. This transition is particularly important for localized cancer therapy, where biomaterials must respond to complex tumor microenvironments while maintaining biocompatibility, controlled release, and site-specific activity.

Stimuli-responsive smart polymers and nanostructures can change their physicochemical properties in response to physical, chemical, or biological cues [209,210]. Internal triggers such as acidic pH, redox imbalance, enzymes, and hypoxia can be used to activate drug release within tumor tissue, while external triggers such as temperature, light, ultrasound, and magnetic fields provide additional spatiotemporal control [206,209]. These responsive features improve tumor specificity, reduce premature release, and minimize systemic toxicity.

Future systems are expected to move from single-stimulus platforms toward multi-responsive biomaterials that integrate several triggers in one design. For example, a nanocarrier or hydrogel may remain stable during circulation, localize within tumor tissue, expose targeting agents in response to pH or enzymes, and release its payload after redox, thermal, or light activation. Such sequential responsiveness links biomaterial structure, stimulus sensitivity, release kinetics, and therapeutic performance. AI and ML can accelerate this development by predicting stimulus thresholds, response kinetics, phase transitions, and structure-property relationships [211]. These tools can help optimize biomaterial composition, crosslinking density, surface charge, degradation rate, and drug-release behavior, thereby reducing experimental trial-and-error [206,212].

### 5.5. Integration with Tissue Repair

Another future direction for localized cancer therapy is combining tumor killing with tissue repair, particularly after surgical resection, where residual cancer cells must be eliminated while damaged tissue is regenerated. Functional biomaterials can support this dual goal by integrating controlled drug release, immune modulation, scaffold-guided regeneration, and patient-specific structural support.

AI and ML are increasingly advancing regenerative medicine by supporting iPSC-related workflows, scaffold design, graft-outcome prediction, and donor-matching strategies, while also improving stem cell classification tasks, for which reported accuracies often exceed 90% [213]. In biomaterial-guided repair, AI and ML can predict cell–biomaterial interactions, optimize scaffold composition and architecture, and improve 3D bioprinting while reducing experimental time and cost [208]. These tools are especially useful for cancer-repair biomaterials, where porosity, stiffness, degradation, bioactive cues, and drug-release kinetics must be balanced to promote healing while inhibiting tumor recurrence.

AI-assisted modeling and digital twins that integrate biological and mechanical may further enable outcome prediction and patient-specific simulation of scaffold performance before clinical use [208,214]. Overall, AI-guided modeling, scaffold optimization, and personalized regenerative strategies can help advance localized cancer therapy from conventional drug-releasing implants toward multifunctional biomaterials that combine cancer control with functional tissue restoration [215].

### 5.6. Limitations and Challenges in Engineering Functional Biomaterials

Despite their promise, engineered functional biomaterials still face major biological, design, and translational barriers. A central limitation is tumor heterogeneity, since cancer type, stage, vascular structure, extracellular matrix density, receptor expression, immune infiltration, and microenvironmental gradients can vary between patients and within the same tumor [10,11,12]. Resistant subpopulations can also survive treatment and contribute to recurrence or progression [13,14]. At the tissue level, abnormal vasculature, elevated interstitial pressure, and stromal barriers restrict drug penetration and produce nonuniform drug exposure [22,23]. These barriers help explain why passive tumor accumulation through the EPR effect remains variable, and why only a small fraction of injected nanomaterials often reaches tumors, while much of the dose is cleared by the liver and spleen or persists in tissues depending on material properties [52,67].

Another challenge is that biomaterial design variables are highly interdependent. Surface charge, PEG density, ligand density, particle size, morphology, porosity, crosslinking density, degradation rate, and stiffness cannot be optimized independently. For example, PEGylation can reduce protein adsorption and macrophage uptake, but excessive shielding may reduce ligand exposure and receptor-mediated uptake [88]. Similarly, active targeting may improve cancer-cell interaction in vitro, but ligand-coated nanoparticles must still overcome serum-protein adsorption, macrophage capture, vascular transport, and extracellular-matrix diffusion before receptor binding can occur [95,96]. In localized systems, increasing porosity or reducing crosslinking may improve diffusion and release, but can compromise mechanical stability or predictable degradation [104,106]. Therefore, biomaterial optimization should be treated as an integrated SPP problem rather than a single-parameter design.

Clinical translation also remains difficult because multifunctional biomaterials introduce challenges in reproducibility, storage stability, biodegradation, long-term safety, and regulatory classification. Natural polymers may offer favorable biocompatibility but can show batch variability, whereas synthetic systems provide better compositional control but require careful safety validation [39,216]. Safety evaluation should therefore extend beyond short-term cytotoxicity assays to include inflammation, immune activation, hemocompatibility, protein-corona-mediated changes in biological identity, degradation-product toxicity, biodistribution, clearance pathways, and long-term tissue accumulation under clinically relevant dosing conditions [217]. This is particularly important for multifunctional or slowly degradable biomaterials because particle size, surface chemistry, degradation behavior, and organ distribution can influence macrophage uptake, renal/hepatobiliary clearance, material persistence, and cumulative tissue exposure [218]. Biomimetic, exosome-based, living, and biohybrid systems offer stronger biological functionality, but their translation is limited by manufacturing complexity, quality control, heterogeneity, and maintenance of biological activity [48,53,62]. Although AI and ML can support formulation screening and predictive design, their reliability depends on standardized, high-quality experimental datasets and strong experimental validation [58]. Addressing these challenges will require more clinically realistic testing models, standardized characterization, scalable manufacturing protocols, and closer integration of materials engineering, pharmacology, and clinical oncology.

## 6. Conclusions

Functional biomaterials are advancing cancer drug delivery by enabling localized, targeted, sustained, and stimulus-responsive therapy. The relevance lies in addressing key limitations of conventional treatment, including poor tumor selectivity, rapid clearance, systemic toxicity, inadequate tumor penetration, and postoperative recurrence. This review shows that biomaterial performance is best understood through a structure–property–performance framework, where material design features directly influence transport, biological interaction, release behavior, degradation, and therapeutic outcome.

In this work, we elucidated how, at the nanoscale, particle size, shape, and surface chemistry regulate circulation, renal clearance, mononuclear phagocyte system uptake, tumor accumulation, penetration, and cellular internalization. Surface modification strategies such as PEGylation, protein-corona control, charge switching, and ligand functionalization can improve delivery, but their effectiveness depends on the ability to overcome biological barriers before receptor-mediated uptake occurs. Therefore, active targeting should be viewed as one component of an integrated delivery sequence rather than a standalone solution.

For localized delivery systems, porosity, internal architecture, mechanical stability, and degradation behavior control drug loading, diffusion, burst release, and sustained release. Hydrogels, scaffolds, mesoporous nanoparticles, and layered implants demonstrate that therapeutic performance depends on balancing transport efficiency with structural integrity and degradation kinetics. Thus, increasing porosity may improve release and tissue interaction, but can also compromise stability, structural integrity, or manufacturability.

Discussion of cancer-specific applications further confirms that biomaterial design must be matched to disease biology. Breast cancer, glioblastoma, colorectal cancer, pancreatic cancer, melanoma, ovarian cancer, bladder cancer, and bone tumors each present distinct barriers or challenges, including receptor heterogeneity, blood–brain barrier restriction, immune suppression, surgical recurrence, and tissue-defect repair. Successful systems therefore integrate multiple functions, structures, and tumor biology for specific targeting, remodeling, immune activation, controlled release, and regeneration.

Future development should move toward mechanism-guided and data-assisted biomaterial design. AI and machine learning, personalized formulation strategies, image-guided delivery, smart multi-responsive systems, and cancer-repair biomaterials can improve design precision and clinical success. However, translation will require attention to scalable manufacturing, long-term safety, regulatory classification, and clinically realistic testing models. Overall, functional biomaterials will have the greatest impact when they are engineered as integrated therapeutic systems in which structure, surface interface, architecture, and chemistry are coordinated to improve cancer treatment outcomes.

## Figures and Tables

**Table 1:** Structure–property–performance summary for functional biomaterials.

Design Parameter	Mechanistic/Quantitative Relation	Functional Outcome	Therapeutic Implication
Particle size	Stokes–Einstein relation, Equation (1); diffusivity decreases as diameter increases.	Regulates diffusion, renal clearance, MPS uptake, tumor penetration, and retention.	Balances circulation, accumulation, penetration, and clearance.
Membrane wrapping	Critical wrapping radius, Equation (2); adhesion must overcome membrane bending/tension. λ ≈ 50 nm.	Determines whether membrane wrapping is energetically favorable.	Guides size, adhesion strength, and surface design for uptake.
Receptor-mediated uptake	Wrapping-time relation, Equation (3); wrapping time scales with particle radius squared.	Controls receptor recruitment and endocytic rate.	Improves active targeting and intracellular delivery.
Particle aspect ratio	Aspect-ratio relation, Equation (4); anisotropy controls orientation and wrapping kinetics.	Alters contact area, curvature, rotation, and internalization.	Tunes shape for transport, retention, or uptake.
Surface charge	Zeta-potential/charge-reversal design rule; near-neutral favors stealth, positive charge favors uptake.	Controls colloidal stability, protein adsorption, and corona formation.	Enables stealth-to-active delivery and reduced off-target clearance.
PEG density	Flory radius/mushroom-to-brush transition; chain spacing controls PEG shielding.	Regulates steric shielding, corona formation, macrophage uptake, and ligand exposure.	Prolongs circulation while preserving controlled targeting.
Stimuli-responsive chemistry	Trigger-responsive activation.	Controls PEG shedding, charge reversal, bond cleavage, and release timing.	Improves site-specific release and lowers systemic toxicity.
Pore size	IUPAC pore classification; micropores ≤ 2 nm, mesopores 2–50 nm, macropores > 50 nm.	Controls loading, surface area, fluid entry, and diffusion distance.	Tunes loading capacity, release rate, and local exposure.
Pore connectivity	Effective diffusion relation, Equation (5); connected porosity increases diffusion, while tortuosity reduces it.	Regulates effective diffusivity, permeability, and sustained release.	Supports predictable local and sustained delivery.
Tissue transport from implants	Fick diffusion–elimination relation, Equation (6); concentration depends on tissue diffusion and first-order clearance.	Determines local concentration gradients and penetration distance.	Guides implant spacing, dose, and release duration.
Mechanical stiffness	Gibson–Ashby relation, Equation (7); stiffness decreases as porosity increases.	Controls scaffold/implant integrity, injectability, and tissue compatibility.	Balances mechanical support, local retention, and tissue integration.

**Table 2:** Design goals and trade-offs linking selected design parameters to therapeutic performance of functional biomaterials in localized cancer drug delivery.

Design Parameter	Therapeutic Goal	Advantages	Limitations/Trade-Offs
Pore/channel size	Tune loading and release	Controls storage space, diffusion path, and fluid access	Optimal size depends on drug, matrix, tissue, and release target
Interconnected pores	Improve loading and transport	Increase fluid entry, pore volume, and effective diffusion	Porosity alone may not predict release if pores are disconnected or tortuous
High tortuosity/constricted pores	Prolong release	Increase diffusion resistance and slow drug escape	May cause incomplete release or uneven drug distribution
Hydrogel nanoscale mesh, ~5–500 nm	Regulate drug/protein diffusion	Controls release of dissolved drugs and proteins	Large molecules may need swelling or degradation before release
Hydrogel macropores, ~10–500 µm	Support cell/tissue interaction	Facilitate cell migration and biological integration	Larger pores may weaken depot structure or alter release
Scaffold porosity	Support integration and delivery	Enhances fluid movement and tissue infiltration	High porosity reduces solid fraction and stiffness
Internal surface chemistry	Improve drug loading/retention	Molecules or amine groups tune drug loading	High surface area helps only with favorable drug-surface interactions
Layered architecture	Separate burst and sustained release	Layer position enables early dose followed by prolonged release	More complex fabrication and sequence-dependent performance
Slower degradation/greater stability	Maintain depot function	Preserves scaffold/implant integrity and prolongs release	May slow drug availability and prolong material persistence
Faster degradation/greater fluid access	Accelerate release and clearance	Enlarge transport pathways during treatment	May weaken structure and shorten release duration

## Data Availability

No new experimental data were created in this study. Data sharing is not applicable to this article.
